# Sex-Specific Neurodevelopmental Outcomes Among Offspring of Mothers With SARS-CoV-2 Infection During Pregnancy

**DOI:** 10.1001/jamanetworkopen.2023.4415

**Published:** 2023-03-23

**Authors:** Andrea G. Edlow, Victor M. Castro, Lydia L. Shook, Sebastien Haneuse, Anjali J. Kaimal, Roy H. Perlis

**Affiliations:** 1 Division of Maternal-Fetal Medicine, Department of Obstetrics and Gynecology, Massachusetts General Hospital and Harvard Medical School, Boston; 2 Center for Quantitative Health, Massachusetts General Hospital, Boston; 3 Research Information Science and Computing, Mass General Brigham, Somerville, Massachusetts; 4 Department of Biostatistics, Harvard T. H. Chan School of Public Health, Boston, Massachusetts; 5 Department of Obstetrics and Gynecology, University of South Florida College of Medicine, Tampa; 6 Department of Psychiatry, Massachusetts General Hospital and Harvard Medical School, Boston

## Abstract

**Importance:**

Prior studies using large registries have suggested a modest increase in risk for neurodevelopmental diagnoses among children of mothers with immune activation during pregnancy, and such risk may be sex-specific.

**Objective:**

To determine whether in utero exposure to SARS-CoV-2 is associated with sex-specific risk for neurodevelopmental disorders up to 18 months after birth, compared with unexposed offspring born during or prior to the COVID-19 pandemic period.

**Design, Setting, and Participants:**

This retrospective cohort study included the live offspring of all mothers who delivered between January 1 and December 31, 2018 (born and followed up before the COVID-19 pandemic), between March 1 and December 31, 2019 (born before and followed up during the COVID-19 pandemic), and between March 1, 2020, and May 31, 2021 (born and followed up during the COVID-19 pandemic). Offspring were born at any of 8 hospitals across 2 health systems in Massachusetts.

**Exposures:**

Polymerase chain reaction evidence of maternal SARS-CoV-2 infection during pregnancy.

**Main Outcomes and Measures:**

Electronic health record documentation of *International Statistical Classification of Diseases and Related Health Problems, Tenth Revision* diagnostic codes corresponding to neurodevelopmental disorders.

**Results:**

The COVID-19 pandemic cohort included 18 355 live births (9399 boys [51.2%]), including 883 (4.8%) with maternal SARS-CoV-2 positivity during pregnancy. The cohort included 1809 Asian individuals (9.9%), 1635 Black individuals (8.9%), 12 718 White individuals (69.3%), and 1714 individuals (9.3%) who were of other race (American Indian or Alaska Native, Native Hawaiian or other Pacific Islander, more than 1 race); 2617 individuals (14.3%) were of Hispanic ethnicity. Mean maternal age was 33.0 (IQR, 30.0-36.0) years. In adjusted regression models accounting for race, ethnicity, insurance status, hospital type (academic center vs community), maternal age, and preterm status, maternal SARS-CoV-2 positivity was associated with a statistically significant elevation in risk for neurodevelopmental diagnoses at 12 months among male offspring (adjusted OR, 1.94 [95% CI 1.12-3.17]; *P* = .01) but not female offspring (adjusted OR, 0.89 [95% CI, 0.39-1.76]; *P* = .77). Similar effects were identified using matched analyses in lieu of regression. At 18 months, more modest effects were observed in male offspring (adjusted OR, 1.42 [95% CI, 0.92-2.11]; *P* = .10).

**Conclusions and Relevance:**

In this cohort study of offspring with SARS-CoV-2 exposure in utero, such exposure was associated with greater magnitude of risk for neurodevelopmental diagnoses among male offspring at 12 months following birth. As with prior studies of maternal infection, substantially larger cohorts and longer follow-up will be required to reliably estimate or refute risk.

## Key Points

**Question:**

Is in utero exposure to maternal SARS-CoV-2 infection associated with greater rates of neurodevelopmental disorder diagnoses in male or female offspring, compared with controls with no such exposure?

**Findings:**

This cohort study of 18 355 infants delivered after February 2020 found that male but not female offspring born to mothers with a positive SARS-CoV-2 polymerase chain reaction test result during pregnancy were more likely to receive a neurodevelopmental diagnosis in the first 12 months after delivery, even after accounting for preterm delivery.

**Meaning:**

These findings suggest that male offspring exposed to SARS-CoV-2 in utero may be at increased risk for neurodevelopmental disorders.

## Teaser

This cohort study examines the risk for neurodevelopmental disorders among male and female offspring exposed in utero to SARS-CoV-2 and whether pregnancy during the COVID-19 pandemic is associated with neurodevelopmental risk independent of SARS-CoV-2 exposure.

## Introduction

Multiple epidemiologic studies using large-scale administrative or health registry data sets suggest that maternal infection or immune activation during pregnancy is associated with risk for a range of neurodevelopmental disorders among offspring. Two early studies—one using data from Helsinki, Finland,^1^ another using national data from Denmark^2^—both identified an association between pregnancy during periods of influenza exposure and schizophrenia hospitalization in offspring. A study of similar design using UK data from the 1957 influenza pandemic^3^ likewise identified elevated rates of schizophrenia among offspring. However, all 3 studies relied on birth dates relative to pandemic activity, rather than examining documented maternal infection, raising the possibility that some other aspect of pregnancy during the pandemic period could confer risk.

More recent investigations have directly examined the association between offspring neurodevelopmental risk and maternal infection outside pandemic periods. A Swedish study of 2.3 million births^4^ found a 30% increase in risk for autism spectrum disorder among offspring of women with an inpatient infection diagnosis during pregnancy. A subsequent analysis of 1.8 million births in Sweden^5^ identified a 79% increase in risk for autism spectrum disorder diagnosis in offspring and a 24% increase in risk of major depression following any maternal infection, without evidence of an effect of infection severity. Animal model studies^6,7,8^ have demonstrated that viral and bacterial infection in pregnancy, as well as noninfectious maternal immune activation, are associated with a range of neurodevelopmental morbidities in offspring.

In light of the magnitude of the COVID-19 pandemic, there is a critical public health need to understand the extent to which maternal exposure may have similar effects on offspring to those observed in these prior studies of infection in pregnancy. Findings of neuropsychiatric sequelae of SARS-CoV-2 infection among both adults and children raise concern that, even absent direct infection of the central nervous system, SARS-CoV-2 may exert persistent effects on the brain.^9,10,11,12,13,14^

Studies of maternal and placental immune response^15^ also suggest sex-specific responses to SARS-CoV-2 infection, with upregulation of placental interferon signaling and reduced maternal anti–SARS-CoV-2 antibodies among male compared with female offspring. Sex-specific responses to maternal exposures have been posited to underlie well-established sex differences in the prevalence of neurodevelopmental and neuropsychiatric disorders like autism, attention-deficit/hyperactivity disorder, anxiety, and major depression.^16,17,18^

Direct investigations of neurodevelopmental diagnoses among SARS-CoV-2–exposed offspring have begun to emerge. A recent meta-analysis including 691 prospectively assessed SARS-CoV-2–exposed offspring evaluated at 3 to 12 months of age^19^ identified no significant increase in a composite measure of neurodevelopmental impairment in SARS-CoV-2–exposed offspring, but did identify a significant increase in the odds of fine motor impairment compared with offspring with no SARS-CoV-2 exposure in utero. One challenge in interpreting such studies has been the absence of contemporaneous comparator groups^20,21^ and the relatively brief follow-up periods. Even for studies that include contemporaneous comparator groups, such as 1 study that suggested elevated neurodevelopmental diagnoses might reflect greater parental stress during the COVID-19 pandemic,^22^ modest sample sizes preclude detection of effects of maternal SARS-CoV-2 infection in the range of other now-accepted neurodevelopmental risks associated with influenza or other maternal infections. Electronic health record (EHR)–based studies are one way to rapidly assemble larger cohorts to address the urgent need for information about potential neurodevelopmental consequences of maternal SARS-CoV-2 infection. We previously reported preliminary evidence of an association between in utero exposure to SARS-CoV-2 and a delay in motor and speech milestones in children at 1 year of age^23^ based on EHR data. Herein, we sought to examine this risk in a cohort 4 times larger, to address 2 questions that could not be explored in the prior study. First, are there sex-specific differences in risk, as might be expected based on evidence of sexual dimorphism in SARS-CoV-2 response and greater male vulnerability to neurodevelopmental insults? Second, was pregnancy during the pandemic, compared with pregnancy prior to the pandemic, associated with neurodevelopmental risk independent of SARS-CoV-2 exposure? We also extended follow-up for a subset of this cohort to estimate whether neurodevelopmental effects were evident by 18 months, rather than 12 months as in prior work.

## Methods

### Study Design and Data Set Generation

In this cohort study, we applied the same cohort definitions as those previously reported.^23^ Specifically, we queried EHRs as represented in the electronic data warehouse spanning a total of 8 hospitals in Eastern Massachusetts, including 2 tertiary care medical centers and 6 community hospitals, as well as all affiliated outpatient networks. Data were extracted for Massachusetts General Hospital, Brigham and Women’s Hospital, Newton-Wellesley Hospital, North Shore Medical Center, Martha’s Vineyard Hospital, Nantucket Cottage Hospital, Cooley Dickinson Hospital, and Wentworth Douglass Hospital, which share a common electronic data warehouse and governance. The Massachusetts General–Brigham Institutional Review Board approved all aspects of this study and allowed a waiver of informed consent because no patient contact was required, the study was considered to be minimal risk, and consent could not feasibly be obtained. This study followed the Strengthening the Reporting of Observational Studies in Epidemiology (STROBE) reporting guideline.

**Figure 1.**
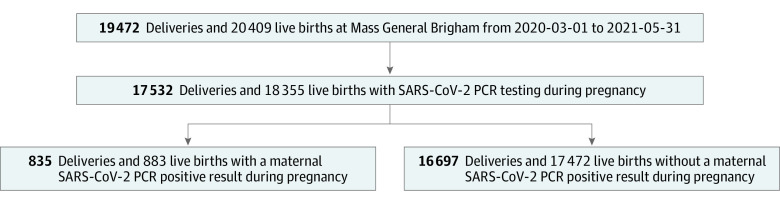
Study Flow Diagram of Pandemic Cohort Derivation PCR indicates polymerase chain reaction.

We began by identifying every live birth in one of these hospitals beginning March 1, 2020, and ending May 31, 2021, representing the COVID-19 pandemic cohort (Figure 1). We generated a new comparison cohort not incorporated in our prior investigation, including all live births between January 1 and December 31, 2018, representing a cohort of children born and followed up prior to the pandemic. For further sensitivity analysis, we also examined a third cohort born prior to the pandemic, but followed up during the pandemic at least in part, spanning all live births between March 1 and December 31, 2019, to evaluate for ascertainment bias that might make physicians more likely to diagnose neurodevelopmental disorders in children during the pandemic. For all cohorts, we linked offspring to the pregnant parent based on medical record number, date and time of birth, and offspring sex. We characterized maternal medical history via *International Statistical Classification of Diseases and Related Health Problems, Tenth Revision* (*ICD-10*) billing codes, problem lists, medications, and laboratory studies occurring between the date of estimated last menstrual period and the discharge date for the delivery admission. SARS-CoV-2 vaccination status was drawn from documentation in the electronic data warehouse, which integrates vaccination orders, reports of vaccine status, and regional public health records. Electronic health records were also used to determine sociodemographic features, including maternal age, self-reported sex, insurance type, and self-reported race (Asian, Black, White, or other [American Indian or Alaska Native, Native Hawaiian or other Pacific Islander, more than 1 race]) and ethnicity (Hispanic or non-Hispanic) to facilitate control for potential confounding variables.

### Outcome Definition

For all offspring, we extracted *ICD-10* billing codes and problem lists. The primary outcome was defined as any diagnosis of a neurodevelopmental disorder within 12 months of birth, or within 18 months of birth for secondary analysis, based on presence of at least 1 *ICD-10* code included in the Healthcare Cost and Utilization Project level 2 developmental category (code 654). These codes included F8x (pervasive and specific developmental disorders: developmental disorders of speech and language [F80]; specific developmental disorders of scholastic skills [F81]; specific developmental disorder of motor function [F82]; pervasive developmental disorders [F84]; other and unspecific disorder of psychological development [F88 and F89]) and F7x (intellectual disabilities). As in our prior work,^23^ we allowed diagnoses that are not typically assigned in the first 18 months of life, to allow for a consistent definition that could be preserved across age groups in future investigations.

### Exposure Definition

Maternal SARS-CoV-2 positivity was defined by the presence of a positive SARS-CoV-2 polymerase chain reaction (PCR) result during the pregnancy and available in the electronic data warehouse. The electronic data warehouse integrates laboratory SARS-CoV-2 PCR results from hospital network laboratories as well as laboratories outside the network. Every birth was linked to the pregnant individual’s test results at any point during pregnancy. Universal screening for SARS-CoV-2 by PCR on admission to labor and delivery departments was implemented across the Massachusetts General–Brigham health systems in April 2020 and continues to the present. Thus, at minimum, asymptomatic pregnant patients were tested at the time of hospitalization. During the study period, patients with positive results of home rapid antigen testing were advised to confirm and/or document a positive home test result with hospital-accessible PCR, per hospital infection control policy. Those individuals with only negative PCR results documented were considered to be negative. Individuals at the beginning of the COVID-19 pandemic who did not receive such testing at admission, or those for whom SARS-CoV-2 test results were not available in the EHR, were excluded from analysis (Figure 1 and eTable 1 in Supplement 1).

### Statistical Analysis

We fit multiple logistic regression models separately among male and female offspring to examine the association between maternal SARS-CoV-2 status and the presence or absence of neurodevelopmental outcome at any point within the first 12 months following birth, adjusting for potential confounding variables associated with either risk for maternal SARS-CoV-2 infection^24^ or neurodevelopmental outcomes in offspring,^25^ including maternal age in years, race and ethnicity, insurance type (public vs private), and hospital type, to yield adjusted estimates of effect and 95% CIs. In these models, SARS-CoV-2–exposed offspring were compared with contemporaneous unexposed controls. Survival analysis was not applied, as 12 months of follow-up were available for all offspring. Primary models also included preterm delivery status, as it is a well-documented risk factor for adverse neurodevelopmental outcomes that has been associated with SARS-CoV-2 positivity during pregnancy.^26,27,28,29^ Thus, primary models sought to examine risk associated with SARS-CoV-2 positivity, after accounting for preterm delivery as well as potentially confounding sociodemographic features; that is, is SARS-CoV-2 positivity associated with greater neurodevelopmental risk than preterm delivery alone? As 18-month outcomes were available for a smaller subset of offspring, neurodevelopmental outcomes among this group were a secondary end point. Nonindependence of multiple births was addressed by considering observations to be clustered within deliveries; the glm.cluster command in the R miceadds package, version 3.11-6 (R Project for Statistical Computing) was used to generate robust standard errors. Sensitivity analyses are described in eMethods in Supplement 1.

To test the hypothesis that there might be greater rates of neurodevelopmental diagnoses among offspring of pandemic-era pregnancies compared with prepandemic pregnancies, we compared this COVID-19 pandemic–born birth cohort (regardless of SARS-CoV-2 exposure status) with 2 different groups. The first included all live births in 2018, allowing for a full 12 months of follow-up prior to the pandemic. The second included all live births beginning in March 2019, recognizing that some follow-up would occur during the pandemic which might itself impact detection of neurodevelopmental outcomes. As in primary analyses, we examined multiple logistic regression models for male and female offspring separately, then pooled both sexes. All analyses used R, version 4.0.3 (R Project for Statistical Computing), with statistical significance defined as uncorrected 2-tailed *P* < .05.

## Results

The analyzable COVID-19 pandemic cohort included 18 355 live births (Figure 1) (9399 boys [51.2%] and 8956 girls [48.8%]). In terms of race, 1809 individuals (9.9%) were Asian, 1635 (8.9%) were Black, 12 718 (69%) were White, 1714 (9.3%) were of other race (American Indian or Alaska Native, Native Hawaiian or other Pacific Islander, or more than 1 race), and 479 (2.6%) were of unknown race; 2617 (14.3%) were of Hispanic ethnicity. Mean maternal age was 33.0 (IQR, 30.0-36.0) years. The cohort included 883 offspring (4.8%) born to mothers with SARS-CoV-2 positivity during pregnancy; the latter group was more likely to be Hispanic (335 [38.0%] vs 2282 [13.1%]), to be Black or of other race (422 [47.8%] vs 4736 [27.1%]), and to have public vs private insurance (427 [48.4%] vs 3166 [18.1%]; *P* < .001 for all) (Table 1), and offspring were more likely to be delivered preterm (117 [13.3%] vs 1740 [10.0%]; *P* = .002). Because the cohort included multiple as well as singleton pregnancies, eTable 2 in Supplement 1 reports characteristics of mothers rather than offspring. Of note, 2054 offspring were excluded because no maternal SARS-CoV-2 test results during pregnancy were available (Figure 1 and eTable 1 in Supplement 1); 1917 (93.3%) of these offspring were delivered before June 2020.

**Table 1.** Sociodemographic and Baseline Clinical Characteristics of Mothers and Offspring

Characteristic	Maternal SARS-CoV-2 status during pregnancy^a^		*P* value^b^
	Negative (n = 17 472)	Positive (n = 883)	
Maternal age, mean (SD), y	33.1 (4.8)	31.3 (5.6)	<.001
Maternal race			
Asian	1762 (10.1)	47 (5.3)	<.001
Black	1496 (8.6)	139 (15.7)	
White	12 307 (70.4)	411 (46.5)	
Other^c^	1478 (8.5)	236 (26.7)	
Unknown	429 (2.5)	50 (5.7)	
Maternal ethnicity^d^			
Hispanic	2282 (13.1)	335 (38.0)	<.001
Non-Hispanic	14 699 (84.1)	514 (58.2)	
Unavailable	483 (2.8)	34 (3.9)	
Maternal public insurance	3166 (18.1)	427 (48.4)	<.001
Trimester of maternal SARS-CoV-2 infection^e^			
First	NA	68 (7.7)	>.99
Second	NA	233 (26.4)	
Third	NA	546 (61.8)	
Delivery hospital type			
Academic medical center	10 185 (58.2)	624 (70.7)	<.001
Community hospital	7287 (41.7)	259 (29.3)	
Delivery method			
Cesarean	5731 (32.8)	300 (34.0)	.47
Vaginal	11 741 (67.2)	583 (66.0)	
Multiple births	1099 (6.3)	71 (8.0)	.04
Delivery admission length of stay, median (IQR), d	3.00 (2.00-4.00)	3.00 (2.00-4.00)	.65
Birth			
Preterm	1740 (10.0)	117 (13.3)	.002
Term	15 732 (90.0)	766 (86.7)	
Offspring sex			
Female	8533 (48.8)	423 (47.9)	.59
Male	8939 (51.2)	460 (52.1)	

Abbreviation: NA, not applicable.

^a^ Unless otherwise indicated, data are expressed as No. (%) of mothers of live offspring. Percentages have been rounded and may not total 100. Data were collected between March 1, 2020, and May 31, 2021.

^b^ Calculated using the Wilcoxon rank sum test, Pearson χ^2^ test, or Fisher exact test.

^c^ Includes American Indian or Alaska Native, Native Hawaiian or other Pacific Islander, and more than 1 race.

^d^ Ethnicity was unknown for 8 mothers in the COVID-19–negative group.

^e^ Trimester of infection was unknown for 36 mothers in the COVID-19–positive group.

Of the 883 SARS-CoV-2–exposed offspring, 26 (2.9%) received a neurodevelopmental diagnosis during the first 12 months of life (this included 0 of 13 whose mothers were partially or fully vaccinated at time of infection) compared with 317 (1.8%) among the SARS-CoV-2–unexposed offspring; counts of specific neurodevelopmental diagnostic codes, which may occur more than once per individual, are listed in eTable 2 in Supplement 1. Three SARS-CoV-2–unexposed children, and none of the exposed children, died prior to 12 months of follow-up and were excluded a priori from analysis.

**Figure 2.**
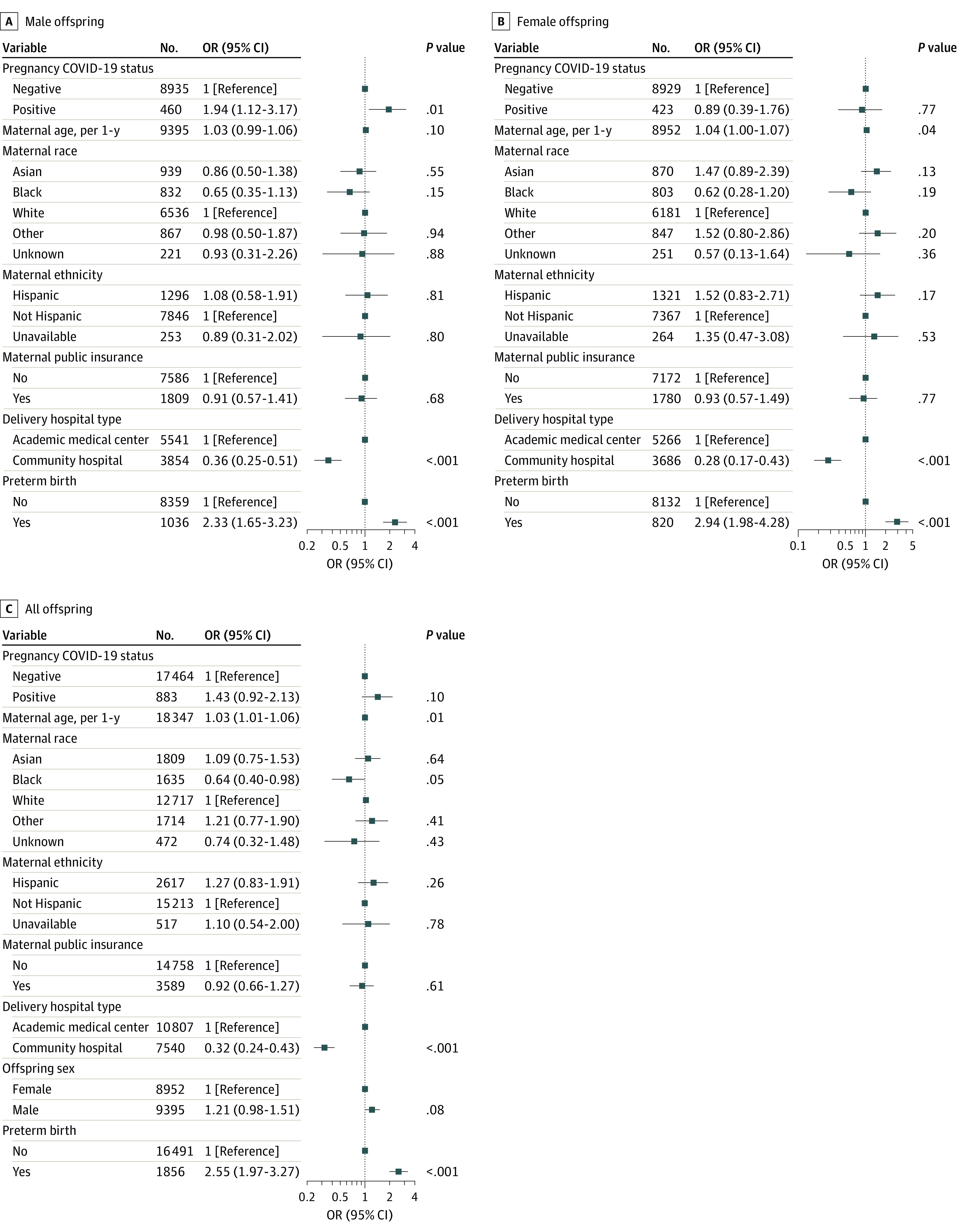
Multiple Logistic Regression Forest Plot of Neurodevelopmental Diagnosis Within 12 Months Offspring are stratified by sex (A and B) and pooled (C). Adjusted regression models accounted for maternal age, maternal race and ethnicity, maternal insurance type, delivery hospital type, and preterm birth in association with maternal SARS-CoV-2 positivity.

In regression models adjusted for race, ethnicity, insurance status, hospital type, maternal age, and preterm status, a statistically significant elevation in risk associated with maternal SARS-CoV-2 positivity was observed among male offspring (adjusted OR, 1.94 [95% CI, 1.12-3.17]; *P* = .01) (Figure 2A) but not female offspring (adjusted OR, 0.89 [95% CI, 0.39-1.76]; *P* = .77) (Figure 2B). In pooled analysis, maternal SARS-CoV-2 positivity was associated with numerically but not statistically significantly greater rates of neurodevelopmental diagnoses (adjusted OR, 1.43 [95% CI, 0.92-2.13]; *P* = .10) (Figure 2C). To generate estimates of association with SARS-CoV-2 exposure inclusive of the effects of preterm delivery, we repeated these analyses without covarying for preterm delivery (eFigure 1 in Supplement 1) with similar results: adjusted ORs were 1.99 (95% CI, 1.15-3.25; *P* = .009) for male offspring, 0.95 (95% CI, 0.42-1.87; *P* = .89) for female offspring, and 1.49 (95% CI, 0.96-2.22; *P* = .06) for pooled offspring. Sensitivity analyses including logistic regression models restricted to offspring with at least 1 follow-up visit at 12 or 18 months, exact matching on the same features as regression models, and coarsened exact matching all supported the robustness of the primary analysis (eResults, eTables 4 and 5, and eFigure 2 in Supplement 1).

We also considered the potential for misclassification bias^30^ to affect estimates of association, if a lack of sufficient availability of SARS-CoV-2 PCR testing led some offspring who were actually exposed in utero to be misclassified as unexposed. Without adjustment, the OR for association between exposure and 12-month neurodevelopmental diagnosis among male offspring was 1.64 (95% CI, 1.09-2.46). Setting PCR sensitivity to 70% (ie, if 30% of true-positive results were not detected), misclassification-adjusted OR was 2.11 (95% CI, 1.21-3.70); at a sensitivity of 50%, the OR was 2.16 (95% CI, 1.21-3.86).

**Figure 3.**
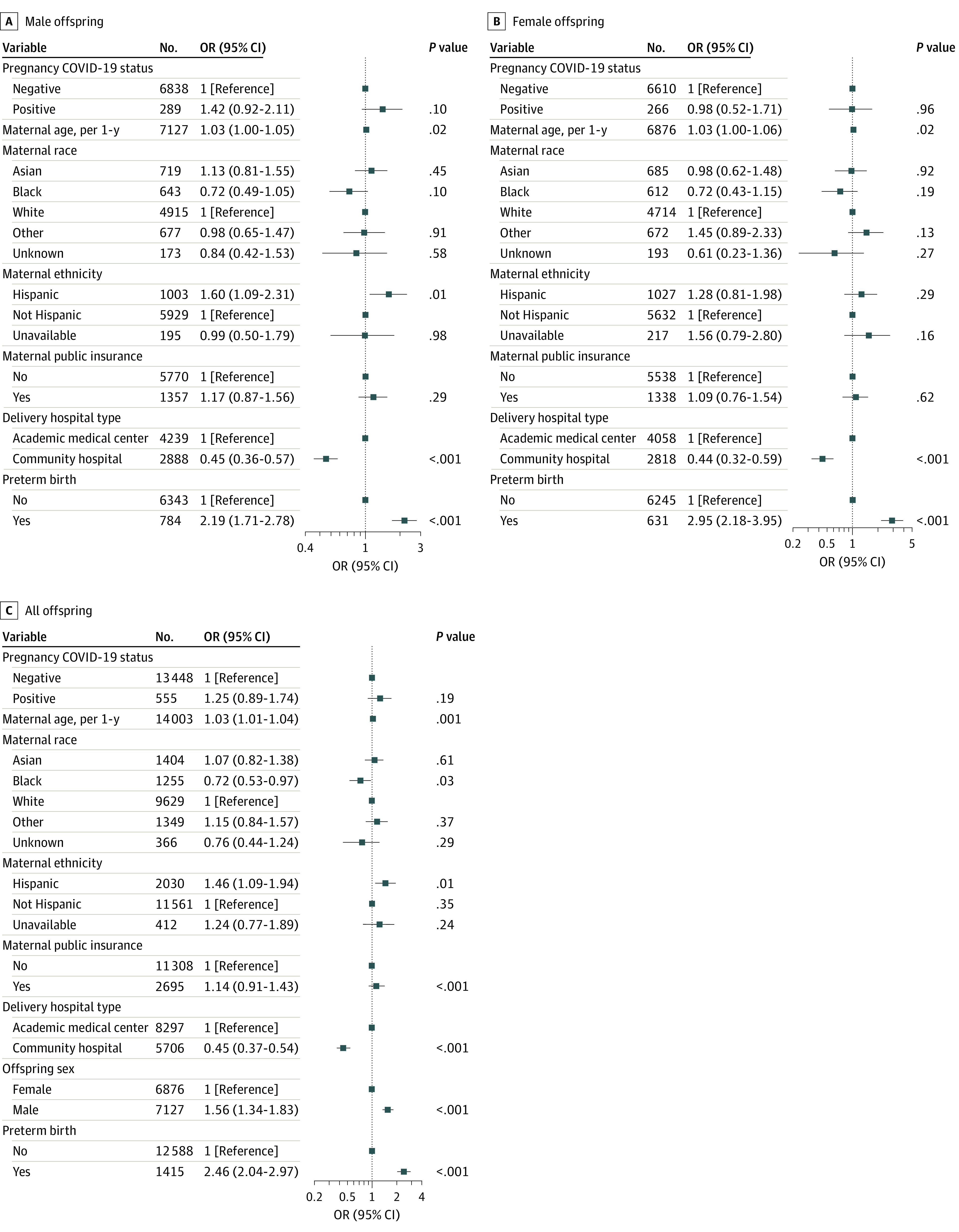
Multiple Logistic Regression Forest Plot of Neurodevelopmental Diagnosis Within 18 Months Offspring are stratified by sex (A and B) and pooled (C). Adjusted regression models accounted for maternal age, maternal race and ethnicity, maternal insurance type, delivery hospital type, and preterm birth in association with maternal SARS-CoV-2 positivity.

We next examined outcomes at 18 months, which were available for 13 452 SARS-CoV-2–unexposed and 555 SARS-CoV-2–exposed offspring (Table 2, and eTable 2 in Supplement 1). Of the 555 SARS-CoV-2–exposed offspring, 43 (7.7%) received a neurodevelopmental diagnosis during the first 18 months of life, compared with 666 (5.0%) among the SARS-CoV-2–unexposed offspring; counts of specific neurodevelopmental diagnostic codes are listed in eTable 2 in Supplement 1. As with 12-month outcomes, greater numeric magnitude of risk was observed among male offspring (adjusted OR, 1.42 [95% CI, 0.92-2.11]; *P* = .10) but not female offspring (adjusted OR, 0.98 [95% CI, 0.52-1.71]; *P* = .96), with all 95% CIs including 1 (Figure 3A and B). In sex-pooled analysis, SARS-CoV-2 exposure was associated with a numeric but not statistically significant increase in neurodevelopmental outcomes (OR, 1.25 [95% CI, 0.89-1.74]; *P* = .19) (Figure 3C). Omitting preterm delivery as a covariate again yielded similar results (eFigure 3A-C in Supplement 1). Finally, as with 12-month outcomes, in sensitivity analysis including only offspring with at least 1 visit in our hospital system at 18 months (eTable 6 in Supplement 1) (218 SARS-CoV-2–exposed and 4609 SARS-CoV-2–unexposed offspring, including 43 of 218 [19.7%] and 610 of 4609 [13.2%], respectively, with a neurodevelopmental diagnosis), we observed effect estimates of similar magnitude to the prior analyses (adjusted ORs, 1.45 [0.91-2.25; *P* = .10] for male offspring; 0.99 [0.50-1.79; *P* > .99] for female offspring; and 1.27 [0.87-1.81; *P* = .20] for pooled offspring) (eFigure 4 in Supplement 1).

**Table 2.** Sociodemographic and Baseline Clinical Characteristics of Mothers and Offspring With 18-Month Follow-up Data

Characteristic	Maternal SARS-CoV-2 status during pregnancy^a^		*P* value^b^
	Negative (n = 13 452)	Positive (n = 555)	
Maternal age, mean (SD), y	33.0 (4.9)	31.1 (5.8)	<.001
Maternal race			
Asian	1375 (10.2)	29 (5.2)	<.001
Black	1153 (8.6)	102 (18.4)	
White	9399 (69.9)	231 (41.6)	
Other^c^	1188 (8.8)	161 (29.0)	
Unknown	337 (2.5)	32 (5.8)	
Maternal ethnicity^d^			
Hispanic	1796 (13.4)	234 (42.2)	<.001
Non-Hispanic	11 266 (83.7)	295 (53.2)	
Unavailable	386 (2.9)	26 (4.7)	
Maternal public insurance	2411 (17.9)	286 (51.5)	<.001
Trimester of maternal SARS-CoV-2 infection^e^			
First	NA	46 (8.3)	>.99
Second	NA	114 (20.5)	
Third	NA	386 (69.5)	
Unknown	NA	9 (1.6)	
Delivery hospital type			
Academic medical center	7901 (58.7)	397 (71.5)	<.001
Community hospital	5551 (41.3)	158 (28.5)	
Delivery method			
Cesarean	4456 (33.1)	190 (34.2)	.59
Vaginal	8996 (66.9)	365 (65.8)	
Multiple births	843 (6.3)	46 (8.3)	.056
Delivery admission length of stay, median (IQR), d	3.00 (2.00-4.00)	3.00 (2.00-4.00)	.63
Birth			
Preterm	1333 (9.9)	82 (14.8)	<.001
Term	12 119 (90.1)	473 (85.2)	
Offspring sex			
Female	6613 (49.2)	266 (47.9)	.57
Male	6839 (50.8)	289 (52.1)	

Abbreviation: NA, not applicable.

^a^ Unless otherwise indicated, data are expressed as No. (%) of mothers of live offspring. Percentages have been rounded and may not total 100. Data were collected between March 1, 2020, and May 31, 2021.

^b^ Calculated using the Wilcoxon rank sum test, Pearson χ^2^ test, or Fisher exact test.

^c^ Includes American Indian or Alaska Native, Native Hawaiian or other Pacific Islander, and more than 1 race or ethnicity.

^d^ Ethnicity was unknown for 4 mothers in the COVID-19–negative group.

^e^ Trimester of infection was unknown for 9 mothers in the COVID-19–positive group.

We also tested for the presence of secular trends in the diagnosis of neurodevelopmental disorders by comparing 12-month outcomes among children born during the COVID-19 pandemic (regardless of maternal SARS-CoV-2 status) with 2 prepandemic cohorts. These analyses demonstrated a modest but not statistically significant increase in rates of neurodevelopmental diagnoses in COVID-19 pandemic–born children relative to children born in 2018 and followed up before the pandemic (eResults, eTables 7-9, and eFigure 5 in Supplement 1) and a statistically significant increase in neurodevelopmental diagnoses in pooled analyses of male and female COVID-19 pandemic–born children, relative to children born before the pandemic (2019) but followed up during the pandemic (adjusted OR for analyses pooling both sexes, 1.24 [95% CI, 1.05-1.47]; *P* = .01) (eFigure 6 in Supplement 1).

## Discussion

Among a cohort of 18 355 infants born during the COVID-19 pandemic with 12 months of follow-up data, we identified a statistically significant elevation in risk among male but not female offspring, detectable using 2 complementary approaches to address potential confounding variables. These effects were not attributable to preterm delivery, which was significantly more common among SARS-CoV-2–exposed offspring. Restriction of the cohort to offspring with 12-month follow-up visits within our hospital system yielded results of similar magnitude, suggesting they are less likely to be a consequence of differential follow-up among exposed offspring. In the smaller subset of offspring for whom 18 months have elapsed since birth, we observed a similar pattern, with likelihood of a neurodevelopmental diagnosis numerically greater among SARS-CoV-2–exposed male offspring, although the 95% CI for this association included 1.00. The similar pattern of increased neurodevelopmental risk among the 18-month cohort but the 95% CI spanning 1.00 suggests the importance of assembling larger cohorts that can be followed up over longer periods of time to determine whether the increased risk noted at 12 months persists.

As all deliveries occurred during the COVID-19 pandemic period, these effects cannot be attributed primarily to nonspecific effects of pandemic-era stress, given that both SARS-CoV-2–positive and uninfected mothers were exposed to such stress. While further work is needed to understand the mechanism by which maternal infection associates with sex-specific risk, differential impact of maternal infection and immune activation on male vs female immune and inflammatory signaling has been noted in prior work.^31,32,33,34^ Our findings are consistent with abundant evidence that the developing male brain is more vulnerable to in utero environmental effects,^35,36^ including maternal immune activation.^37^ In the case of SARS-CoV-2 infection in pregnancy, prior work identified robust immune activation at the maternal-fetal interface, even in the absence of placental infection and vertical transmission.^15,38,39^ Moreover, this work demonstrated sexually dimorphic effects of maternal SARS-CoV-2 positivity on placental immune response, with significant upregulation of types I, II, and III interferon signaling pathways in male placentas and downregulation in female placentas.^15^ Consistent with these findings in human SARS-CoV-2 infection, in murine models, upregulation of maternal type I interferon signaling in pregnancy was noted to be associated with a type I interferon signature in the embryonic yolk sac, a preplacental structure that serves as the origin of fetal brain microglia, and reduced microglial proliferation in offspring.^40^ These changes, in turn, were associated with a sex-specific impact on offspring behavior, with exposed male offspring demonstrating increased anxiety relative to female offspring.^40^ Thus, our finding that neurodevelopment in male offspring may be more adversely impacted than in female offspring in the setting of maternal SARS-CoV-2 infection is biologically plausible and consistent with prior studies that have demonstrated sex-specific impact of maternal types I, II, and III interferon signaling on the developing placenta and fetal brain.^15,40^

We did detect an increase in the risk for neurodevelopmental diagnoses, more modest in magnitude and similar among male and female offspring, associated with pandemic-era deliveries compared with 2018 (a prepandemic cohort with all offspring having 12-month follow-up prior to the COVID-19 pandemic) and 2019 (a prepandemic-born cohort with all offspring having at least some follow-up during the COVID-19 pandemic). The increased odds of any neurodevelopmental diagnosis at 12 months in pandemic-born children was statistically significant in a pooled analysis of both sexes, in comparison with the prepandemic 2019 cohort. These comparisons with historical controls suggest that any secular trends (reflecting birth during the pandemic itself) are likely to have a more modest impact on observed neurodevelopmental risk in offspring than does maternal SARS-CoV-2 infection itself.

Overall, our results are consistent with abundant evidence that exposure to infection during pregnancy—including viral infections such as influenza—is associated with an increased risk for neurodevelopmental morbidity in offspring.^5,41,42,43,44,45^ Such risk was initially detected as an increase in schizophrenia and autism spectrum disorder diagnoses following influenza and rubella pandemics,^1,2,3^ was recapitulated in animal models,^8,46,47^ and was more recently directly tested in large registry studies.^4,5^ Risk of behavioral dysfunction in offspring after maternal prenatal exposures also has been reported to be greater in male offspring in rodent models.^32,48^ Because the neurodevelopmental risk in offspring is thought to be mediated in large part through maternal and placental immune activation^6,7,8^ and has been observed in other viral infections that, similar to SARS-CoV-2, are not thought to directly infect fetal brain tissue,^6^ it is biologically plausible that SARS-CoV-2 infection in pregnancy could impact offspring risk for neurodevelopmental disorders.

Prior investigations of neurodevelopment among SARS-CoV-2-exposed offspring yielded mixed results. A recent meta-analysis^19^ included 11 438 infants screened with the Ages and Stages Questionnaire–3 (ASQ-3)^49^ up to 12 months of age, of whom 691 had documented SARS-CoV-2 exposure. Of note, time to follow-up was not uniform among the included studies, and studies were included that assessed infants once at ages ranging from 3 to 12 months. That analysis found no significant difference in overall rate of a composite neurodevelopmental impairment score combining all ASQ-3 domains among SARS-CoV-2–exposed offspring (12% among SARS-CoV-2–exposed vs 9% in contemporaneous SARS-CoV-2–unexposed offspring; OR, 1.38 [95% CI, 0.80-2.37]; *P* = .24). However, the meta-analysis did identify significantly greater rates of fine motor impairment in offspring exposed to maternal SARS-CoV-2 in utero (OR, 3.46 [95% CI, 1.43-8.38]; *P* = .006). Some individual studies, including an early 3-month longitudinal study of mothers in China^20^ and a 10- to 12-month prospective study in Kuwait,^21^ have been limited by the absence of a contemporaneous noninfected control group. An early study of 57 SARS-CoV-2–exposed offspring in China that did include controls did not identify adverse outcomes at 3 months.^50^ Such a control group may be particularly important in light of another small 6-month study,^22^ including 114 SARS-CoV-2–exposed offspring, that failed to identify risk associated with exposure per se. That study did identify elevated scores among pandemic-era vs prepandemic offspring,^22^ suggesting a secular trend that was posited to reflect greater stress among parents during the COVID-19 pandemic, although the study did not assess parental stress.

Conversely, a preliminary report in a subset of the present cohort, including a noninfected contemporary control group, suggested that SARS-CoV-2 was associated with increased risk for neurodevelopmental diagnosis at 12 months of age,^23^ but did not yet have sufficient neurodevelopmental diagnoses to examine the impact of fetal sex, as we have now been able to examine in this substantially larger cohort. As such, the present study does not refute our prior work,^23^ but suggests the previously observed effect was either driven by a sex-specific increase in neurodevelopmental risk, and/or that there is greater heterogeneity of effect among female offspring. This observation may still represent type I error, and further study will be required to more precisely understand the magnitude of risk that exists, if any.

We detected a more modest elevation in risk associated with birth during the COVID-19 pandemic period. We emphasize that such an effect cannot explain the SARS-CoV-2 associations we observe, as primary analyses are limited to the pandemic period. There are instead a wealth of potential explanations for such a secular trend, many unrelated to biology. While greater maternal stress could represent one such explanation, as posited by a recent small study,^22^ absent direct measures of stress, this conclusion is purely speculative. Other potential explanations for this finding that are unrelated to maternal biology could include ascertainment bias (eg, clinicians being more likely to diagnose neurodevelopmental disorders in children born during the pandemic), changes in social environment for offspring during the pandemic, or even changes in billing as health systems adapt to remote assessment. These alternate explanations merit investigation in future studies.

### Limitations

This study has some limitations. While it expands the size of our prior cohort 4-fold,^23^ we emphasize the importance of larger-scale investigation with longer-term follow-up to understand the potential neurodevelopmental risks and to investigate key questions related to trimester of maternal SARS-CoV-2 infection and to maternal vaccination status. It is not clear that the changes we can detect at 12 and 18 months will be indicative of persistent risks for disorders such as autism spectrum disorder, intellectual disability, or schizophrenia. For this reason, systematic prospective neuropsychiatric phenotyping in children with in utero exposure will be particularly important to complement and extend our results.

Some of the potential biases due to missing data could increase the likelihood of a null finding. For example, some mothers with no documented SARS-CoV-2 infection could actually have had asymptomatic infection or an inability to access testing during illness. While universal screening and the standardized infection control practices referenced should ensure accuracy of the record for SARS-CoV-2 infection in pregnancy, it is certainly possible that some patients with only a negative PCR result available at delivery were positive at an earlier point in pregnancy that was not captured with a PCR test; such undetected positivity among our control population would be more likely to bias our results toward the null. Our rates of maternal positivity, while lower than those in New York City early in the pandemic, are otherwise consistent with those presented in a recent review,^51^ as well as in a prior report reflecting surveillance at admission during this period.^52^

In an analysis of quantitative bias associated with misclassification, we find that decreasing the apparent sensitivity of SARS-CoV-2 testing as a means of modeling true infections not detected by the available data would yield substantially greater estimates of association than those we report. Finally, although data about vertical transmission were not uniformly available in this current EHR cohort due to hospital neonatal screening practices that changed throughout the pandemic, vertical transmission was likely a rare event. Population-level estimates of vertical transmission range from 1% to 3%,^53^ with rates lower than 1% reported in multiple studies.^54,55^

Children who were evaluated outside of the outpatient pediatric networks of these 8 hospitals could have received neurodevelopmental diagnoses not documented in follow-up pediatric notes. In the case of neurodevelopmental outcomes, results could be biased if mothers with SARS-CoV-2 infection during pregnancy received closer follow-up, increasing the likelihood of detection of any diagnosis in this group. In our prior work,^23^ we have shown that other diagnoses are not inflated, as would be expected if children of previously ill mothers were more likely to be evaluated or receive more frequent pediatric assessment. Moreover, in sensitivity analyses limited to offspring who had documented visits within our health care system at 12 months, we observed effects of similar magnitude to those in the full cohort. Further discussion of collider bias as it relates to COVID-19 can be found in Griffith et al.^56^ Nonetheless, these limitations underscore the importance of multiple, complementary follow-up study designs, rather than reliance on any single investigation or methodology.

## Conclusions

The findings of this EHR-based cohort study suggest that male children exposed to maternal SARS-CoV-2 infection in utero may be at modestly greater risk for adverse neurodevelopmental outcomes. This study further suggests a small secular trend of increased neurodevelopmental diagnoses in children born during the pandemic compared with prepandemic births, independent of maternal infection status. The latter may reflect a form of ascertainment bias, with clinicians more likely to be attuned to neurodevelopmental diagnoses in children born during the pandemic. In aggregate, our results underscore the need for larger-scale studies applying a range of follow-up strategies to more precisely estimate and characterize risk.

## Notes

Supplement 1. **eMethods.** Analysis **eResults.** Sensitivity Analyses **eTable 1.** Comparison of Features of Offspring by Availability of Maternal SARS-CoV-2 PCR Results During Pregnancy **eTable 2.** Neurodevelopmental Diagnostic Codes Observed Among SARS-CoV-2 Exposed and Unexposed Offspring at 12 Months (Top) and 18 Months (Bottom) **eTable 3.** Sociodemographic and Baseline Clinical Characteristics of Mothers Who Were SARS-CoV-2 Positive or Negative Included in the Pandemic Cohort (March 2020 to May 2021) **eTable 4.** Sociodemographic and Baseline Clinical Characteristics of Offspring of Mothers Who Were SARS-CoV-2 Positive or Negative Included in the Pandemic Cohort (March 2020 to May 2021), Restricted to Offspring With at Least 1 Follow-up Visit at 12 Months ± 60 Days **eTable 5.** Coarsened Exact Matching of Mothers With or Without SARS-CoV-2 Exposure **eTable 6.** Sociodemographic and Baseline Clinical Characteristics of Offspring With Mothers Who Were SARS-CoV-2 Positive or Negative Included in the Pandemic Cohort (March 2020 to May 2021), Restricted to Offspring With at Least 1 Follow-up Visit at 18 Months ± 60 Days **eTable 7.** Sociodemographic and Baseline Clinical Characteristics of Offspring Who Were Born During the Pandemic (March 2020 to May 2021), Compared With Those Born in 2018 **eTable 8.** Neurodevelopmental Diagnostic Codes of Offspring Who Were Born During the Pandemic (March 2020 to May 2021), Compared With Those Born in 2018 or 2019 **eTable 9.** Sociodemographic and Baseline Clinical Characteristics of Offspring Who Were Born During the Pandemic (March 2020 to May 2021), Compared With Those Born in 2019 **eFigure 1.** Multiple Logistic Regression Forest Plot of Neurodevelopmental Diagnosis Within 12 Months in Offspring Associated With Maternal COVID-19 Status, Without Adjustment for Preterm Birth **eFigure 2.** Multiple Logistic Regression Forest Plot of Neurodevelopmental Diagnosis Within 12 Months in Offspring Associated With Maternal COVID-19 Status, With Cohort Restricted to Offspring With at Least 1 Follow-up Visit at 12 Months ± 60 Days **eFigure 3.** Multiple Logistic Regression Forest Plot of Neurodevelopmental Diagnosis Within 18 Months in Offspring Associated With Maternal COVID-19 Status, Without Adjustment for Preterm Birth **eFigure 4.** Multiple Logistic Regression Forest Plot of Neurodevelopmental Diagnosis Within 18 Months in Offspring Associated With Maternal COVID-19 Status, With Cohort Restricted to Offspring With at Least 1 Follow-up Visit at 18 Months ± 60 Days **eFigure 5.** Multiple Logistic Regression Forest Plot of Neurodevelopmental Diagnosis Within 12 Months in Offspring Associated With Period of Birth (Pandemic vs 2018) **eFigure 6.** Multiple Logistic Regression Forest Plot of Neurodevelopmental Diagnosis Within 12 Months in Offspring Associated With Period of Birth (Pandemic vs 2019)

Supplement 2.
Data Sharing Statement
