# Supplementary material for: Sex-Specific Neurodevelopmental Outcomes Among Offspring of Mothers With SARS-CoV-2 Infection During Pregnancy
Source: JAMA Netw Open. 2023 Mar 23;6(3):e234415. doi: 10.1001/jamanetworkopen.2023.4415 (PMC10037162; doi:10.1001/jamanetworkopen.2023.4415)
Supplement: Supplement 1. — eMethods. Analysis eResults. Sensitivity Analyses eTable 1. Comparison of Features of Offspring by Availability of Maternal SARS-CoV-2 PCR Results During Pregnancy eTable 2. Neurodevelopmental Diagnostic Codes Observed Among SARS-CoV-2 Exposed and Unexposed Offspring at 12 Months (Top) and 18 Months (Bottom) eTable 3. Sociodemographic and Baseline Clinical Characteristics of Mothers Who Were SARS-CoV-2 Positive or Negative Included in the Pandemic Cohort (March 2020 to May 2021) eTable 4. Sociodemographic and Baseline Clinical Characteristics of Offspring of Mothers Who Were SARS-CoV-2 Positive or Negative Included in the Pandemic Cohort (March 2020 to May 2021), Restricted to Offspring With at Least 1 Follow-up Visit at 12 Months ± 60 Days eTable 5. Coarsened Exact Matching of Mothers With or Without SARS-CoV-2 Exposure eTable 6. Sociodemographic and Baseline Clinical Characteristics of Offspring With Mothers Who Were SARS-CoV-2 Positive or Negative Included in the Pandemic Cohort (March 2020 to May 2021), Restricted to Offspring With at Least 1 Follow-up Visit at 18 Months ± 60 Days eTable 7. Sociodemographic and Baseline Clinical Characteristics of Offspring Who Were Born During the Pandemic (March 2020 to May 2021), Compared With Those Born in 2018 eTable 8. Neurodevelopmental Diagnostic Codes of Offspring Who Were Born During the Pandemic (March 2020 to May 2021), Compared With Those Born in 2018 or 2019 eTable 9. Sociodemographic and Baseline Clinical Characteristics of Offspring Who Were Born During the Pandemic (March 2020 to May 2021), Compared With Those Born in 2019 eFigure 1. Multiple Logistic Regression Forest Plot of Neurodevelopmental Diagnosis Within 12 Months in Offspring Associated With Maternal COVID-19 Status, Without Adjustment for Preterm Birth eFigure 2. Multiple Logistic Regression Forest Plot of Neurodevelopmental Diagnosis Within 12 Months in Offspring Associated With Maternal COVID-19 Status, With Cohort Restricted to Offspring With at Lea [file jamanetwopen-e234415-s001.pdf]

Supplementary Online Content

Edlow AG, Castro VM, Shook LL, Haneuse S, Kaimal AJ, Perlis RH. Sex-specific neurodevelopmental outcomes among offspring of mothers with SARS-CoV-2 infection during pregnancy. *JAMA Netw Open*. 2023;6(3):e234415.
doi:10.1001/jamanetworkopen.2023.4415

eMethods. Analysis

eResults. Sensitivity Analyses

eTable 1. Comparison of Features of Offspring by Availability of Maternal SARS-CoV-2 PCR Results During Pregnancy

eTable 2. Neurodevelopmental Diagnostic Codes Observed Among SARS-CoV-2 Exposed and Unexposed Offspring at 12 Months (Top) and 18 Months (Bottom)

eTable 3. Sociodemographic and Baseline Clinical Characteristics of Mothers Who Were SARS-CoV-2 Positive or Negative Included in the Pandemic Cohort (March 2020 to May 2021)

eTable 4. Sociodemographic and Baseline Clinical Characteristics of Offspring of Mothers Who Were SARS-CoV-2 Positive or Negative Included in the Pandemic Cohort (March 2020 to May 2021), Restricted to Offspring With at Least 1 Follow-up Visit at 12 Months ± 60 Days

eTable 5. Coarsened Exact Matching of Mothers With or Without SARS-CoV-2 Exposure

eTable 6. Sociodemographic and Baseline Clinical Characteristics of Offspring With Mothers Who Were SARS-CoV-2 Positive or Negative Included in the Pandemic Cohort (March 2020 to May 2021), Restricted to Offspring With at Least 1 Follow-up Visit at 18 Months ± 60 Days

eTable 7. Sociodemographic and Baseline Clinical Characteristics of Offspring Who Were Born During the Pandemic (March 2020 to May 2021), Compared With Those Born in 2018

eTable 8. Neurodevelopmental Diagnostic Codes of Offspring Who Were Born During the Pandemic (March 2020 to May 2021), Compared With Those Born in 2018 or 2019

© 2023 Edlow AG et al. *JAMA Network Open*.

eTable 9. Sociodemographic and Baseline Clinical Characteristics of Offspring Who Were Born During the Pandemic (March 2020 to May 2021), Compared With Those Born in 2019

eFigure 1. Multiple Logistic Regression Forest Plot of Neurodevelopmental Diagnosis Within 12 Months in Offspring Associated With Maternal COVID-19 Status, Without Adjustment for Preterm Birth

eFigure 2. Multiple Logistic Regression Forest Plot of Neurodevelopmental Diagnosis Within 12 Months in Offspring Associated With Maternal COVID-19 Status, With Cohort Restricted to Offspring With at Least 1 Follow-up Visit at 12 Months ± 60 Days

eFigure 3. Multiple Logistic Regression Forest Plot of Neurodevelopmental Diagnosis Within 18 Months in Offspring Associated With Maternal COVID-19 Status, Without Adjustment for Preterm Birth

eFigure 4. Multiple Logistic Regression Forest Plot of Neurodevelopmental Diagnosis Within 18 Months in Offspring Associated With Maternal COVID-19 Status, With Cohort Restricted to Offspring With at Least 1 Follow-up Visit at 18 Months ± 60 Days

eFigure 5. Multiple Logistic Regression Forest Plot of Neurodevelopmental Diagnosis Within 12 Months in Offspring Associated With Period of Birth (Pandemic vs 2018)

eFigure 6. Multiple Logistic Regression Forest Plot of Neurodevelopmental Diagnosis Within 12 Months in Offspring Associated With Period of Birth (Pandemic vs 2019)

This supplementary material has been provided by the authors to give readers additional information about their work.

© 2023 Edlow AG et al. *JAMA Network Open*.

eMethods. Analysis

In sensitivity analyses, we repeated the primary outcome analyses confining the analyses to only those offspring who had a 12-month visit within our hospital system (with a 60-day window before or after the 12-month mark), to understand whether associations could be confounded by differential in-person follow-up among exposed or unexposed offspring. We also performed a sensitivity analysis including only offspring with an 18-month visit within our hospital system, again with a 60-day window before and after the 18-month mark.

To examine the robustness of multiple regression results by applying an alternative approach to potential confounding variables, we also applied exact matching to match each SARS-CoV-2-exposed offspring with at least one SARS-CoV-2-unexposed offspring on the same features used in regression models (i.e., race, ethnicity, maternal age, insurance type, hospital type, and preterm delivery). Where regression models estimate an average treatment effect, the matched cohort provides an approximation of the average treatment (i.e., exposure) effect among exposed individuals. We used the R *matchit* (v4.4.0) package for these analyses, allowing as many exact matches among controls as available – i.e., 1:n matching – to maximize precision in estimates of effect. Allowing more than 1 match for each exposed case where available (rather than 1:1) allows greater power to detect association given the larger available pool of controls. To quantify the effect of misclassification of exposure on estimates of association[30], we used the R *episensr* package (v1.1.0).

eResults. Sensitivity Analyses

In sensitivity analyses, we also fit logistic regression models with the cohort restricted to offspring with at least one follow-up visit within our hospital system at 12 months (Supplemental Table 4; n=383 SARS-CoV-2 exposed and 6635 unexposed, including 26/383 (6.8%) and 309/6635 (4.7%) with a neurodevelopmental diagnosis, respectively). These models supported the robustness of the primary analysis, yielding results of similar magnitude to those of the full cohort: adjusted OR was 1.76 (1.00-2.94), p=.04 for males, 0.82 (0.36-1.66, p=.61) for females, and 1.31 (0.83-1.99, p=.22) for both sexes combined (Supplemental Figures 2A, B, and C).

To further examine the robustness of these associations, we used exact matching on the same features as regression – maternal age, race, ethnicity, insurance status; hospital type; and preterm status—to examine the association between offspring SARS-CoV-2 exposure and neurodevelopmental diagnosis. Among males, matching 397 COVID-exposed offspring with 6055 COVID-unexposed offspring, OR for any neurodevelopmental disorder at 12 months was 1.88 (95% CI 1.05-3.37; p=.04), and for female offspring matching 382 COVID-exposed to 5803 COVID-unexposed, OR was 1.22 (0.51-2.47, p=.6). For the cohort as a whole, matching 779 COVID-exposed offspring with 11858 COVID-unexposed offspring, OR was 1.56 (95% CI 0.95-2.41; p=.06). Applying coarsened exact matching rather than exact matching did not was consistent with initial results; see Supplemental Table 5. These sensitivity analyses demonstrated consistent direction of effect across both matched analyses and logistic regression models.

We also tested for the presence of secular trends in diagnosis of neurodevelopmental disorders by comparing 12-month outcomes among children born during the pandemic to two pre-pandemic cohorts. These analyses were all conducted based on birth during versus pre-pandemic, and did not take into account maternal SARS-CoV-2 status. Compared to 2018 (Supplemental Table 7, all children followed up pre-pandemic), a modest but not statistically significant increase in rates of neurodevelopmental diagnoses

© 2023 Edlow AG et al. *JAMA Network Open*.

(Supplemental Table 8) was identified among males (adjusted OR 1.11, 95% CI 0.89-1.39), p=.37) as well as females (adjusted OR 1.28, 95% CI 0.99-1.66), p=.06); analyses pooling both sexes also failed to meet statistical significance adjusted OR=1.18, 95% CI 0.99-1.39, p=.06; Supplemental Figures 5A, B, and C). Compared to the 2019 pre-pandemic cohort (Supplemental Table 9), all children born pre-pandemic but all with 12-month follow-up intra-pandemic), for children born during the pandemic, adjusted OR for neurodevelopmental diagnoses at 12 months among male offspring was 1.24, 95% CI 0.99-1.56, p=.06; for females, 1.26, 95% CI 0.98-1.62, p=.08; analyses pooling both sexes, 1.24, 95% CI 1.05-1.47, p=.01 (Supplemental Figures 6A, B, and C).

© 2023 Edlow AG et al. *JAMA Network Open*.

eTable 1. Comparison of Features of Offspring by Availability of Maternal SARS-CoV-2 PCR Results During Pregnancy

Characteristic	SARS-COV-2 PCR available N=18355	SARS-COV-2 PCR not available N=2054	p-value[1]
**Delivery hospital type, n (%)**			0.36
Academic medical center	10,809 (59)	1,231 (60)	
Community hospital	7,546 (41)	823 (40)	
**Maternal age, Median (IQR)**	33.0 (30.0 – 36.0)	33.0 (30.0 – 36.0)	0.24
**Maternal race, n (%)**			0.44
Asian	1,809 (9.9)	216 (11)	
Black or African American	1,635 (8.9)	184 (9.0)	
Other	1,714 (9.3)	186 (9.1)	
Unknown	479 (2.6)	66 (3.2)	
White	12,718 (69)	1,402 (68)	
**Maternal ethnicity, n (%)**			0.12
Hispanic	2,617 (14)	284 (14)	
Not Hispanic	15,213 (83)	1,695 (83)	
Unavailable	517 (2.8)	74 (3.6)	
Unknown	8	1	
**Offspring sex, n (%)**			0.24
Female	8,956 (49)	1,030 (50)	
Male	9,399 (51)	1,024 (50)	
**Maternal public insurance, n (%)**	3,593 (20)	376 (18)	0.17
**Delivery method, n (%)**			0.30
C-Section	6,031 (33)	698 (34)	
Vaginal	12,324 (67)	1,356 (66)	
**Pre-term birth, n (%)**			0.93
Preterm	1,857 (10)	209 (10)	
Term	16,498 (90)	1,845 (90)	
**Delivery admission length of stay (days), Median (IQR)**	3.00 (2.00 – 4.00)	3.00 (2.00 – 4.00)	0.026
**Multiple births, n (%)**	1,170 (6.4)	156 (7.6)	0.033
**Pandemic delivery period, n (%)**			<0.001
2020-1 Mar - May 2020	2,139 (12)	1,917 (93)	
2020-2 Jun - Aug 2020	4,411 (24)	20 (1.0)	
2020-3 Sep - Nov 2020	3,926 (21)	20 (1.0)	
2020-4 Dec 2020 - Feb 2021	3,522 (19)	25 (1.2)	
2021-5 Mar - May 2021	4,357 (24)	72 (3.5)	

[1]Pearson's Chi-squared test; Wilcoxon rank sum test

© 2023 Edlow AG et al. *JAMA Network Open*.

eTable 2. Neurodevelopmental Diagnostic Codes Observed Among SARS-CoV-2 Exposed and Unexposed Offspring at 12 Months (Top) and 18 Months (Bottom)

ICD-10-CM code	ICD-10-CM description	Pregnancy SARS-COV-2 Negative			Pregnancy SARS-COV-2 Positive		
		All offspring	Male offspring	Female offspring	All offspring	Male offspring	Female offspring
F82	Specific developmental disorder of motor function	175	85	90	20	12	8
F80.9	Developmental disorder of speech and language, unspecified	75	52	23	5	<5	<5
F89	Unspecified disorder of psychological development	80	44	36	<5	<5	<5
F80.1	Expressive language disorder	42	32	10	<5	<5	0
F88	Other disorders of psychological development	13	9	<5	<5	<5	<5
F80.2	Mixed receptive-expressive language disorder	<5	<5	<5	<5	<5	0
F80.0	Phonological disorder	<5	0	<5	0	0	0
F80.4	Speech and language development delay due to hearing loss	<5	<5	0	0	0	0
F80.89	Other developmental disorders of speech and language	<5	<5	0	0	0	0

ICD-10-CM code	ICD-10-CM description	Pregnancy SARS-COV-2 Negative			Pregnancy SARS-COV-2 Positive		
		All offspring	Male offspring	Female offspring	All offspring	Male offspring	Female offspring
F80.1	Expressive language disorder	248	168	80	9	7	<5
F80.9	Developmental disorder of speech and language, unspecified	211	144	67	23	15	8
F82	Specific developmental disorder of motor function	219	104	115	21	13	8
F89	Unspecified disorder of psychological development	103	58	45	<5	<5	<5
F88	Other disorders of psychological development	17	11	6	<5	<5	<5
F80.2	Mixed receptive-expressive language disorder	13	9	<5	<5	<5	0
F80.89	Other developmental disorders of speech and language	<5	<5	0	0	0	0
F80.4	Speech and language development delay due to hearing loss	<5	<5	0	0	0	0
F80.0	Phonological disorder	<5	<5	<5	0	0	0
F81.9	Developmental disorder of scholastic skills, unspecified	<5	<5	0	0	0	0

© 2023 Edlow AG et al. *JAMA Network Open*.

eTable 3. Sociodemographic and Baseline Clinical Characteristics of Mothers Who Were SARS-CoV-2 Positive or Negative Included in the Pandemic Cohort (March 2020 to May 2021)

**Characteristic**	**Pregnancy SARS-COV-2 Negative** N=16715	**Pregnancy SARS-COV-2 Positive** N=835	**p-value**[1]
**Maternal age, Median (IQR)**	33.0 (30.0 – 36.0)	31.0 (27.0 – 35.0)	<0.001
**Maternal race, n (%)**			<0.001
Asian	1,716 (10)	44 (5.3)	
Black or African American	1,427 (8.5)	128 (15)	
Other	1,434 (8.6)	227 (27)	
Unknown	417 (2.5)	44 (5.3)	
White	11,721 (70)	392 (47)	
**Maternal ethnicity, n (%)**			<0.001
Hispanic	2,212 (13)	328 (39)	
Not Hispanic	14,029 (84)	480 (57)	
Unavailable	466 (2.8)	27 (3.2)	
Unknown	8	0	
**Maternal public insurance, n (%)**	3,081 (18)	415 (50)	<0.001
**Delivery hospital type, n (%)**			<0.001
Academic medical center	9,651 (58)	583 (70)	
Community hospital	7,064 (42)	252 (30)	
**Delivery method, n (%)**			0.66
C-Section	5,243 (31)	268 (32)	
Vaginal	11,472 (69)	567 (68)	
**Delivery admission length of stay (days), Median (IQR)**	3.00 (2.00 – 3.00)	3.00 (2.00 – 4.00)	0.68
**Pre-term birth, n (%)**			0.024
Preterm	1,300 (7.8)	83 (9.9)	
Term	15,415 (92)	752 (90)	

1Wilcoxon rank sum test; Pearson's Chi-squared test
Table 1 in the main text reports offspring characteristics, while this table reports features of mothers.

© 2023 Edlow AG et al. *JAMA Network Open*.

eTable 4. Sociodemographic and Baseline Clinical Characteristics of Offspring of Mothers Who Were SARS-CoV-2 Positive or Negative Included in the Pandemic Cohort (March 2020 to May 2021), Restricted to Offspring With at Least 1 Follow-up Visit at 12 Months ± 60 Days

Characteristic	No Pediatric Follow-up Visit at 12-months +/- 60 days N=11337	Pediatric Follow-up Visit at 12-months +/- 60 days N=7018	p-value[1]
**Delivery hospital type, n (%)**			0.55
Academic medical center	6,657 (59)	4,152 (59)	
Community hospital	4,680 (41)	2,866 (41)	
**Maternal age, Mean (SD)**	32.9 (4.9)	33.1 (4.9)	0.004
**Maternal race, n (%)**			<0.001
Asian	1,059 (9.3)	750 (11)	
Black or African American	1,178 (10)	457 (6.5)	
Other	952 (8.4)	762 (11)	
Unknown	291 (2.6)	188 (2.7)	
White	7,857 (69)	4,861 (69)	
**Maternal ethnicity, n (%)**			<0.001
Hispanic	1,492 (13)	1,125 (16)	
Not Hispanic	9,520 (84)	5,693 (81)	
Unavailable	318 (2.8)	199 (2.8)	
Unknown	7	1	
**Offspring sex, n (%)**			0.030
Female	5,603 (49)	3,353 (48)	
Male	5,734 (51)	3,665 (52)	
**Maternal public insurance, n (%)**	2,096 (18)	1,497 (21)	<0.001
**Pre-term birth, n (%)**			<0.001
Preterm	1,078 (9.5)	779 (11)	
Term	10,259 (90)	6,239 (89)	
**Delivery admission length of stay (days), Median (IQR)**	3.00 (2.00 – 4.00)	3.00 (2.00 – 4.00)	0.050
**Multiple births, n (%)**	687 (6.1)	483 (6.9)	0.027

[1]Pearson's Chi-squared test; Wilcoxon rank sum test

© 2023 Edlow AG et al. *JAMA Network Open*.

eTable 5. Coarsened Exact Matching of Mothers With or Without SARS-CoV-2 Exposure

Coarsened exact match analysis	Odds ratio	95% Confidence interval	p-value
Matched offspring - 12m Male	1.83	(0.97-3.16)	0.04
Matched offspring - 12m Female	1.22	(0.51-2.47)	0.62
Matched offspring - 12m ALL	1.56	(0.95-2.41)	0.06

© 2023 Edlow AG et al. *JAMA Network Open*.

eTable 6. Sociodemographic and Baseline Clinical Characteristics of Offspring With Mothers Who Were SARS-CoV-2 Positive or Negative Included in the Pandemic Cohort (March 2020 to May 2021), Restricted to Offspring With at Least 1 Follow-up Visit at 18 Months ± 60 Days

**Characteristic**	**No Pediatric Follow-up Visit at 18-months +/- 60 days** N=9180	**Pediatric Follow-up Visit at 18-months +/- 60 days** N=4827	**p-value**[1]
**Delivery hospital type, n (%)**			<0.001
Academic medical center	5,341 (58)	2,957 (61)	
Community hospital	3,839 (42)	1,870 (39)	
**Maternal age, Mean (SD)**	32.8 (4.9)	33.1 (4.9)	0.002
**Maternal race, n (%)**			<0.001
Asian	895 (9.7)	509 (11)	
Black or African American	926 (10)	329 (6.8)	
Other	792 (8.6)	557 (12)	
Unknown	254 (2.8)	115 (2.4)	
White	6,313 (69)	3,317 (69)	
**Maternal ethnicity, n (%)**			<0.001
Hispanic	1,216 (13)	814 (17)	
Not Hispanic	7,694 (84)	3,867 (80)	
Unavailable	267 (2.9)	145 (3.0)	
Unknown	3	1	
**Offspring sex, n (%)**			0.020
Female	4,574 (50)	2,305 (48)	
Male	4,606 (50)	2,522 (52)	
**Maternal public insurance, n (%)**	1,650 (18)	1,047 (22)	<0.001
**Pre-term birth, n (%)**			0.015
Preterm	886 (9.7)	529 (11)	
Term	8,294 (90)	4,298 (89)	
**Delivery admission length of stay (days), Median (IQR)**	3.00 (2.00 – 4.00)	3.00 (2.00 – 4.00)	0.26
**Multiple births, n (%)**	547 (6.0)	342 (7.1)	0.009

[1]Pearson's Chi-squared test; Wilcoxon rank sum test

© 2023 Edlow AG et al. *JAMA Network Open*.

eTable 7. Sociodemographic and Baseline Clinical Characteristics of Offspring Who Were Born During the Pandemic (March 2020 to May 2021), Compared With Those Born in 2018

**Characteristic**	**Births in 2018** N=13952	**Pandemic births** N=18355	**p-value**[1]
**Delivery hospital type, n (%)**			<0.001
Academic medical center	8,954 (64)	10,809 (59)	
Community hospital	4,998 (36)	7,546 (41)	
**Maternal age, Mean (SD)**	33.0 (4.9)	33.0 (4.9)	0.74
**Maternal race, n (%)**			<0.001
Asian	1,544 (11)	1,809 (9.9)	
Black or African American	1,320 (9.5)	1,635 (8.9)	
Other	1,335 (9.6)	1,714 (9.3)	
Unknown	430 (3.1)	479 (2.6)	
White	9,323 (67)	12,718 (69)	
**Maternal ethnicity, n (%)**			<0.001
Hispanic	2,032 (15)	2,617 (14)	
Not Hispanic	11,284 (81)	15,213 (83)	
Unavailable	636 (4.6)	517 (2.8)	
Unknown	0	8	
**Offspring sex, n (%)**			0.22
Female	6,904 (49)	8,956 (49)	
Male	7,048 (51)	9,399 (51)	
**Maternal public insurance, n (%)**	2,714 (19)	3,593 (20)	0.78
**Delivery method, n (%)**			0.87
C-Section	4,596 (33)	6,031 (33)	
Vaginal	9,356 (67)	12,324 (67)	
**Pre-term birth, n (%)**			0.003
Preterm	1,555 (11)	1,857 (10)	
Term	12,397 (89)	16,498 (90)	
**Delivery admission length of stay (days), Median (IQR)**	3.00 (2.00 – 4.00)	3.00 (2.00 – 4.00)	<0.001
**Multiple births, n (%)**	1,065 (7.6)	1,170 (6.4)	<0.001

[1]Pearson's Chi-squared test; Wilcoxon rank sum test

© 2023 Edlow AG et al. *JAMA Network Open*.

eTable 8. Neurodevelopmental Diagnostic Codes of Offspring Who Were Born During the Pandemic (March 2020 to May 2021), Compared With Those Born in 2018 or 2019

ICD-10CD	Description	Live births in 2018			Live births in 2019		
		All offspring	Male offspring	Female offspring	All offspring	Male offspring	Female offspring
F82	Specific developmental disorder of motor function	108	50	58	118	56	62
F80.9	Developmental disorder of speech and language, unspecified	54	33	21	55	35	20
F80.1	Expressive language disorder	35	25	10	39	23	16
F89	Unspecified disorder of psychological development	23	16	7	23	15	8
F88	Other disorders of psychological development	13	8	5	8	7	<5
F80.0	Phonological disorder	<5	0	<5	<5	<5	0
F80.2	Mixed receptive-expressive language disorder	0	0	0	<5	0	<5
F80.89	Other developmental disorders of speech and language	<5	<5	0	<5	<5	0
F80.4	Speech and language development delay due to hearing loss	<5	<5	<5	<5	<5	0
F80.81	Childhood onset fluency disorder	<5	<5	0	0	0	0

© 2023 Edlow AG et al. *JAMA Network Open*.

eTable 9. Sociodemographic and Baseline Clinical Characteristics of Offspring Who Were Born During the Pandemic (March 2020 to May 2021), Compared With Those Born in 2019

**Characteristic**	**Births in 2019** N=15386	**Pandemic births** N=18355	**p-value**[1]
**Delivery hospital type, n (%)**			<0.001
Academic medical center	9,672 (63)	10,809 (59)	
Community hospital	5,714 (37)	7,546 (41)	
**Maternal age, Mean (SD)**	33.1 (4.9)	33.0 (4.9)	0.30
**Maternal race, n (%)**			<0.001
Asian	1,740 (11)	1,809 (9.9)	
Black or African American	1,367 (8.9)	1,635 (8.9)	
Other	1,510 (9.8)	1,714 (9.3)	
Unknown	464 (3.0)	479 (2.6)	
White	10,305 (67)	12,718 (69)	
**Maternal ethnicity, n (%)**			0.081
Hispanic	2,308 (15)	2,617 (14)	
Not Hispanic	12,610 (82)	15,213 (83)	
Unavailable	462 (3.0)	517 (2.8)	
Unknown	6	8	
**Offspring sex, n (%)**			0.094
Female	7,648 (50)	8,956 (49)	
Male	7,738 (50)	9,399 (51)	
**Delivery Method, n (%)**			0.74
C-Section	5,029 (33)	6,031 (33)	
Vaginal	10,357 (67)	12,324 (67)	
**Pre-term birth, n (%)**			0.005
Preterm	1,701 (11)	1,857 (10)	
Term	13,685 (89)	16,498 (90)	
**Delivery admission length of stay (days), Median (IQR)**	3.00 (2.00 – 4.00)	3.00 (2.00 – 4.00)	<0.001
**Multiple births, n (%)**	1,071 (7.0)	1,170 (6.4)	0.031

[1]Pearson's Chi-squared test; Wilcoxon rank sum test

© 2023 Edlow AG et al. *JAMA Network Open*.

eFigure 1. Multiple Logistic Regression Forest Plot of Neurodevelopmental Diagnosis Within 12 Months in Offspring Associated With Maternal COVID-19 Status, Without Adjustment for Preterm Birth

A. Male offspring

Variable		N	Odds ratio		p
**Pregnancy COVID status**	COVID negative	8935		Reference	
	COVID positive	460		1.99 (1.15, 3.25)	0.009
**Maternal age (years)**		9395		1.03 (1.00, 1.06)	0.061
**Maternal race**	White	6536		Reference	
	Asian	939		0.85 (0.50, 1.36)	0.512
	Black or African American	832		0.66 (0.36, 1.15)	0.167
	Other	867		0.96 (0.50, 1.83)	0.908
	Unknown	221		0.92 (0.31, 2.25)	0.872
**Maternal ethnicity**	Not Hispanic	7846		Reference	
	Hispanic	1296		1.09 (0.59, 1.93)	0.772
	Unavailable	253		0.92 (0.32, 2.08)	0.856
**Maternal public insurance**	No	7586		Reference	
	Yes	1809		0.90 (0.57, 1.40)	0.650
**Delivery hospital type**	Academic medical center	5541		Reference	
	Community hospital	3854		0.33 (0.23, 0.48)	<0.001
			0.5 1 2		

B. Female offspring

Variable		N	Odds ratio		p
**Pregnancy COVID status**	COVID negative	8529		Reference	
	COVID positive	423		0.95 (0.42, 1.87)	0.89
**Maternal age (years)**		8952		1.04 (1.01, 1.08)	0.02
**Maternal race**	White	6181		Reference	
	Asian	870		1.37 (0.81, 2.22)	0.22
	Black or African American	803		0.64 (0.29, 1.24)	0.22
	Other	847		1.54 (0.81, 2.88)	0.18
	Unknown	251		0.59 (0.14, 1.69)	0.39
**Maternal ethnicity**	Not Hispanic	7367		Reference	
	Hispanic	1321		1.48 (0.81, 2.64)	0.19
	Unavailable	264		1.40 (0.48, 3.17)	0.48
**Maternal public insurance**	No	7172		Reference	
	Yes	1780		0.94 (0.58, 1.50)	0.80
**Delivery hospital type**	Academic medical center	5266		Reference	
	Community hospital	3686		0.26 (0.16, 0.40)	<0.001
			0.2 0.5 1 2		

C. All offspring

© 2023 Edlow AG et al. *JAMA Network Open*.

Variable		N	Odds ratio		p
**Pregnancy COVID status**	COVID negative	17464		Reference	
	COVID positive	883		1.49 (0.96, 2.22)	0.064
**Maternal age (years)**		18347		1.04 (1.01, 1.06)	0.004
**Maternal race**	White	12717		Reference	
	Asian	1809		1.05 (0.73, 1.48)	0.775
	Black or African American	1635		0.65 (0.41, 1.00)	0.065
	Other	1714		1.21 (0.77, 1.89)	0.409
	Unknown	472		0.75 (0.33, 1.51)	0.462
**Maternal ethnicity**	Not Hispanic	15213		Reference	
	Hispanic	2617		1.27 (0.83, 1.90)	0.263
	Unavailable	517		1.13 (0.55, 2.04)	0.721
**Maternal public insurance**	No	14758		Reference	
	Yes	3589		0.92 (0.66, 1.26)	0.600
**Delivery hospital type**	Academic medical center	10807		Reference	
	Community hospital	7540		0.30 (0.22, 0.40)	<0.001
**Offspring sex**	Female	8952		Reference	
	Male	9395		1.25 (1.00, 1.55)	0.046

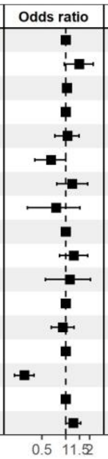

© 2023 Edlow AG et al. *JAMA Network Open*.

eFigure 2. Multiple Logistic Regression Forest Plot of Neurodevelopmental Diagnosis Within 12 Months in Offspring Associated With Maternal COVID-19 Status, With Cohort Restricted to Offspring With at Least 1 Follow-up Visit at 12 Months ± 60 Days

A. Male offspring

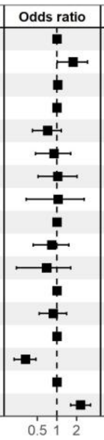

Variable		N	Odds ratio		p
**Pregnancy COVID status**	COVID negative	3459		Reference	
	COVID positive	206		1.76 (1.00, 2.94)	0.04
**Maternal age (years)**		3665		1.02 (0.99, 1.06)	0.21
**Maternal race**	White	2555		Reference	
	Asian	411		0.72 (0.42, 1.17)	0.21
	Black or African American	222		0.90 (0.46, 1.62)	0.74
	Other	389		1.03 (0.51, 2.02)	0.94
	Unknown	88		1.04 (0.34, 2.62)	0.94
**Maternal ethnicity**	Not Hispanic	2991		Reference	
	Hispanic	568		0.83 (0.43, 1.52)	0.55
	Unavailable	106		0.70 (0.24, 1.61)	0.45
**Maternal public insurance**	No	2922		Reference	
	Yes	743		0.87 (0.53, 1.39)	0.58
**Delivery hospital type**	Academic medical center	2159		Reference	
	Community hospital	1506		0.33 (0.22, 0.48)	<0.001
**Pre-term birth**	No	3236		Reference	
	Yes	429		2.31 (1.61, 3.25)	<0.001

B. Female offspring

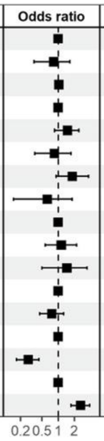

Variable		N	Odds ratio		p
**Pregnancy COVID status**	COVID negative	3175		Reference	
	COVID positive	177		0.82 (0.36, 1.66)	0.61
**Maternal age (years)**		3352		1.03 (0.99, 1.06)	0.16
**Maternal race**	White	2306		Reference	
	Asian	339		1.49 (0.86, 2.44)	0.13
	Black or African American	235		0.85 (0.37, 1.73)	0.68
	Other	373		1.84 (0.91, 3.68)	0.09
	Unknown	99		0.63 (0.15, 1.84)	0.46
**Maternal ethnicity**	Not Hispanic	2702		Reference	
	Hispanic	557		1.15 (0.58, 2.21)	0.69
	Unavailable	93		1.46 (0.49, 3.46)	0.44
**Maternal public insurance**	No	2599		Reference	
	Yes	753		0.77 (0.45, 1.26)	0.30
**Delivery hospital type**	Academic medical center	1993		Reference	
	Community hospital	1359		0.28 (0.17, 0.44)	<0.001
**Pre-term birth**	No	3002		Reference	
	Yes	350		2.61 (1.73, 3.86)	<0.001

C. All offspring

© 2023 Edlow AG et al. *JAMA Network Open*.

Variable		N	Odds ratio		p
**Pregnancy COVID status**	COVID negative	6634		Reference	
	COVID positive	383		1.31 (0.83, 1.99)	0.22
**Maternal age (years)**		7017		1.02 (1.00, 1.05)	0.06
**Maternal race**	White	4861		Reference	
	Asian	750		0.98 (0.67, 1.39)	0.92
	Black or African American	457		0.88 (0.53, 1.39)	0.59
	Other	762		1.36 (0.84, 2.19)	0.21
	Unknown	187		0.81 (0.35, 1.65)	0.60
**Maternal ethnicity**	Not Hispanic	5693		Reference	
	Hispanic	1125		0.97 (0.61, 1.51)	0.89
	Unavailable	199		0.97 (0.47, 1.79)	0.93
**Maternal public insurance**	No	5521		Reference	
	Yes	1496		0.82 (0.58, 1.16)	0.27
**Delivery hospital type**	Academic medical center	4152		Reference	
	Community hospital	2865		0.31 (0.23, 0.41)	<0.001
**Offspring sex**	Female	3352		Reference	
	Male	3665		1.21 (0.97, 1.52)	0.09
**Pre-term birth**	No	6238		Reference	
	Yes	779		2.40 (1.84, 3.11)	<0.001

0.5 1 2

© 2023 Edlow AG et al. *JAMA Network Open*.

eFigure 3. Multiple Logistic Regression Forest Plot of Neurodevelopmental Diagnosis Within 18 Months in Offspring Associated With Maternal COVID-19 Status, Without Adjustment for Preterm Birth

A. Male offspring

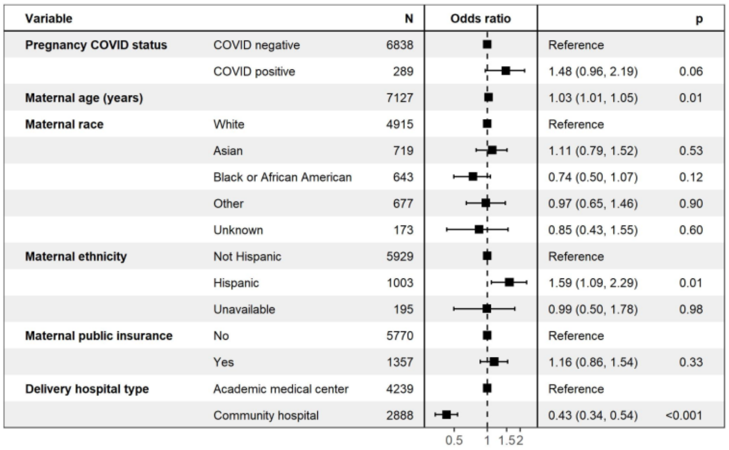

Variable		N	Odds ratio		p
**Pregnancy COVID status**	COVID negative	6838		Reference	
	COVID positive	289		1.48 (0.96, 2.19)	0.06
**Maternal age (years)**		7127		1.03 (1.01, 1.05)	0.01
**Maternal race**	White	4915		Reference	
	Asian	719		1.11 (0.79, 1.52)	0.53
	Black or African American	643		0.74 (0.50, 1.07)	0.12
	Other	677		0.97 (0.65, 1.46)	0.90
	Unknown	173		0.85 (0.43, 1.55)	0.60
**Maternal ethnicity**	Not Hispanic	5929		Reference	
	Hispanic	1003		1.59 (1.09, 2.29)	0.01
	Unavailable	195		0.99 (0.50, 1.78)	0.98
**Maternal public insurance**	No	5770		Reference	
	Yes	1357		1.16 (0.86, 1.54)	0.33
**Delivery hospital type**	Academic medical center	4239		Reference	
	Community hospital	2888		0.43 (0.34, 0.54)	<0.001

B. Female offspring

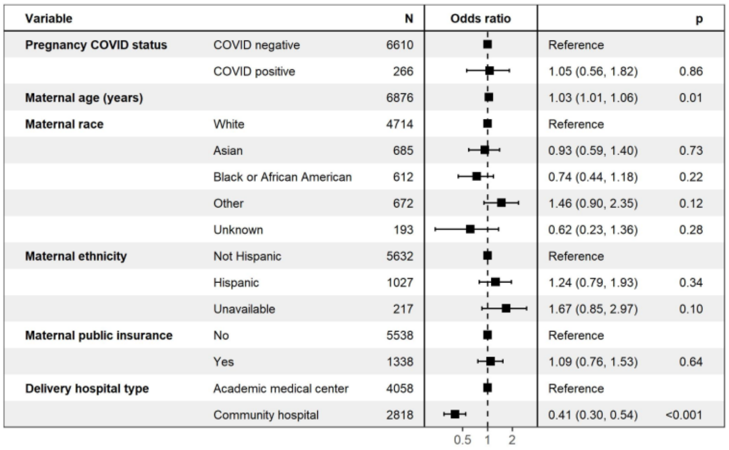

Variable		N	Odds ratio		p
**Pregnancy COVID status**	COVID negative	6610		Reference	
	COVID positive	266		1.05 (0.56, 1.82)	0.86
**Maternal age (years)**		6876		1.03 (1.01, 1.06)	0.01
**Maternal race**	White	4714		Reference	
	Asian	685		0.93 (0.59, 1.40)	0.73
	Black or African American	612		0.74 (0.44, 1.18)	0.22
	Other	672		1.46 (0.90, 2.35)	0.12
	Unknown	193		0.62 (0.23, 1.36)	0.28
**Maternal ethnicity**	Not Hispanic	5632		Reference	
	Hispanic	1027		1.24 (0.79, 1.93)	0.34
	Unavailable	217		1.67 (0.85, 2.97)	0.10
**Maternal public insurance**	No	5538		Reference	
	Yes	1338		1.09 (0.76, 1.53)	0.64
**Delivery hospital type**	Academic medical center	4058		Reference	
	Community hospital	2818		0.41 (0.30, 0.54)	<0.001

C. All offspring

© 2023 Edlow AG et al. *JAMA Network Open*.

Variable		N	Odds ratio		p
**Pregnancy COVID status**	COVID negative	13448		Reference	
	COVID positive	555		1.32 (0.93, 1.82)	0.10
**Maternal age (years)**		14003		1.03 (1.01, 1.05)	<0.001
**Maternal race**	White	9629		Reference	
	Asian	1404		1.04 (0.80, 1.33)	0.78
	Black or African American	1255		0.74 (0.54, 0.99)	0.05
	Other	1349		1.15 (0.84, 1.57)	0.37
	Unknown	366		0.77 (0.45, 1.25)	0.31
**Maternal ethnicity**	Not Hispanic	11561		Reference	
	Hispanic	2030		1.44 (1.08, 1.91)	0.01
	Unavailable	412		1.27 (0.79, 1.92)	0.30
**Maternal public insurance**	No	11308		Reference	
	Yes	2695		1.13 (0.90, 1.41)	0.28
**Delivery hospital type**	Academic medical center	8297		Reference	
	Community hospital	5706		0.42 (0.35, 0.50)	<0.001
**Offspring sex**	Female	6876		Reference	
	Male	7127		1.59 (1.37, 1.87)	<0.001

0.5 1 1.5

© 2023 Edlow AG et al. *JAMA Network Open*.

eFigure 4. Multiple Logistic Regression Forest Plot of Neurodevelopmental Diagnosis Within 18 Months in Offspring Associated With Maternal COVID-19 Status, With Cohort Restricted to Offspring With at Least 1 Follow-up Visit at 18 Months ± 60 Days

A. Male offspring

Variable		N	Odds ratio		p
**Pregnancy COVID status**	COVID negative	2403		Reference	
	COVID positive	119		1.45 (0.91, 2.25)	0.1
**Maternal age (years)**		2522		1.02 (0.99, 1.04)	0.2
**Maternal race**	White	1730		Reference	
	Asian	279		0.98 (0.68, 1.38)	0.9
	Black or African American	161		1.05 (0.67, 1.61)	0.8
	Other	296		0.98 (0.62, 1.55)	0.9
	Unknown	56		1.15 (0.54, 2.29)	0.7
**Maternal ethnicity**	Not Hispanic	2024		Reference	
	Hispanic	426		1.35 (0.88, 2.06)	0.2
	Unavailable	72		0.73 (0.34, 1.41)	0.4
**Maternal public insurance**	No	2000		Reference	
	Yes	522		1.06 (0.76, 1.47)	0.7
**Delivery hospital type**	Academic medical center	1532		Reference	
	Community hospital	990		0.43 (0.33, 0.56)	<0.001
**Pre-term birth**	No	2249		Reference	
	Yes	273		2.27 (1.69, 3.03)	<0.001

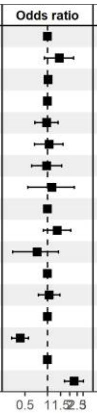

B. Female offspring

Variable		N	Odds ratio		p
**Pregnancy COVID status**	COVID negative	2205		Reference	
	COVID positive	99		0.99 (0.50, 1.79)	1.0
**Maternal age (years)**		2304		1.02 (0.99, 1.05)	0.3
**Maternal race**	White	1587		Reference	
	Asian	230		0.88 (0.53, 1.41)	0.6
	Black or African American	168		0.77 (0.41, 1.33)	0.4
	Other	261		1.51 (0.87, 2.59)	0.1
	Unknown	58		0.67 (0.22, 1.67)	0.4
**Maternal ethnicity**	Not Hispanic	1843		Reference	
	Hispanic	388		1.31 (0.77, 2.19)	0.3
	Unavailable	73		1.54 (0.73, 2.97)	0.2
**Maternal public insurance**	No	1780		Reference	
	Yes	524		0.80 (0.53, 1.18)	0.3
**Delivery hospital type**	Academic medical center	1425		Reference	
	Community hospital	879		0.47 (0.34, 0.65)	<0.001
**Pre-term birth**	No	2048		Reference	
	Yes	256		2.29 (1.62, 3.21)	<0.001

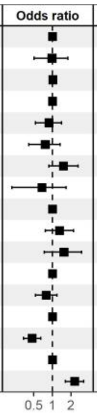

C. All offspring

Variable		N	Odds ratio		p
**Pregnancy COVID status**	COVID negative	4608		Reference	
	COVID positive	218		1.27 (0.87, 1.81)	0.20
**Maternal age (years)**		4826		1.02 (1.00, 1.04)	0.08
**Maternal race**	White	3317		Reference	
	Asian	509		0.94 (0.70, 1.25)	0.70
	Black or African American	329		0.95 (0.66, 1.33)	0.75
	Other	557		1.17 (0.83, 1.67)	0.37
	Unknown	114		0.98 (0.54, 1.70)	0.95
**Maternal ethnicity**	Not Hispanic	3867		Reference	
	Hispanic	814		1.34 (0.96, 1.86)	0.08
	Unavailable	145		1.01 (0.60, 1.62)	0.96
**Maternal public insurance**	No	3780		Reference	
	Yes	1046		0.94 (0.72, 1.21)	0.61
**Delivery hospital type**	Academic medical center	2957		Reference	
	Community hospital	1869		0.45 (0.37, 0.55)	<0.001
**Offspring sex**	Female	2304		Reference	
	Male	2522		1.67 (1.40, 1.98)	<0.001
**Pre-term birth**	No	4297		Reference	
	Yes	529		2.28 (1.82, 2.83)	<0.001

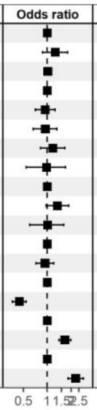

© 2023 Edlow AG et al. *JAMA Network Open*.

eFigure 5. Multiple Logistic Regression Forest Plot of Neurodevelopmental Diagnosis Within 12 Months in Offspring Associated With Period of Birth (Pandemic vs 2018)

A. Male offspring

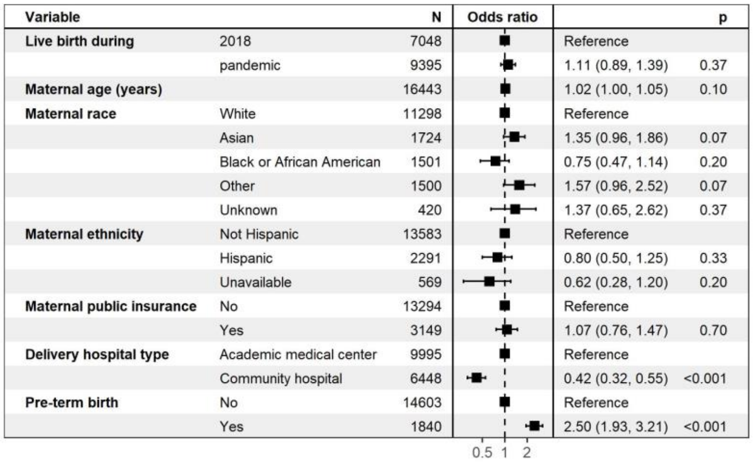

Variable		N	Odds ratio		p
Live birth during	2018	7048		Reference	
	pandemic	9395		1.11 (0.89, 1.39)	0.37
Maternal age (years)		16443		1.02 (1.00, 1.05)	0.10
Maternal race	White	11298		Reference	
	Asian	1724		1.35 (0.96, 1.86)	0.07
	Black or African American	1501		0.75 (0.47, 1.14)	0.20
	Other	1500		1.57 (0.96, 2.52)	0.07
	Unknown	420		1.37 (0.65, 2.62)	0.37
Maternal ethnicity	Not Hispanic	13583		Reference	
	Hispanic	2291		0.80 (0.50, 1.25)	0.33
	Unavailable	569		0.62 (0.28, 1.20)	0.20
Maternal public insurance	No	13294		Reference	
	Yes	3149		1.07 (0.76, 1.47)	0.70
Delivery hospital type	Academic medical center	9995		Reference	
	Community hospital	6448		0.42 (0.32, 0.55)	<0.001
Pre-term birth	No	14603		Reference	
	Yes	1840		2.50 (1.93, 3.21)	<0.001

B. Female offspring

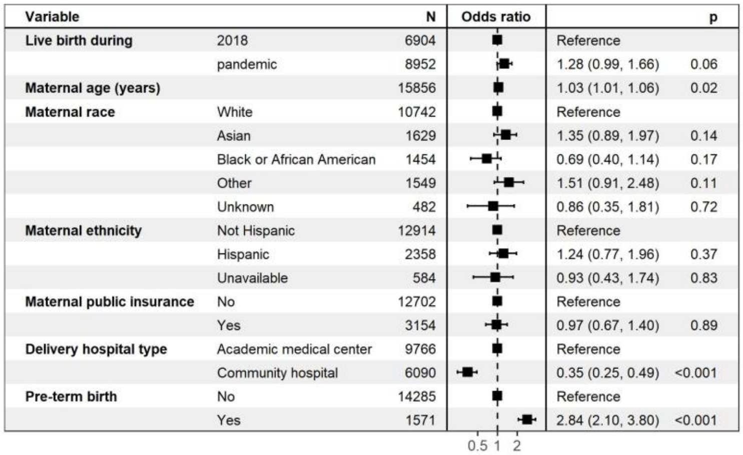

Variable		N	Odds ratio		p
Live birth during	2018	6904		Reference	
	pandemic	8952		1.28 (0.99, 1.66)	0.06
Maternal age (years)		15856		1.03 (1.01, 1.06)	0.02
Maternal race	White	10742		Reference	
	Asian	1629		1.35 (0.89, 1.97)	0.14
	Black or African American	1454		0.69 (0.40, 1.14)	0.17
	Other	1549		1.51 (0.91, 2.48)	0.11
	Unknown	482		0.86 (0.35, 1.81)	0.72
Maternal ethnicity	Not Hispanic	12914		Reference	
	Hispanic	2358		1.24 (0.77, 1.96)	0.37
	Unavailable	584		0.93 (0.43, 1.74)	0.83
Maternal public insurance	No	12702		Reference	
	Yes	3154		0.97 (0.67, 1.40)	0.89
Delivery hospital type	Academic medical center	9766		Reference	
	Community hospital	6090		0.35 (0.25, 0.49)	<0.001
Pre-term birth	No	14285		Reference	
	Yes	1571		2.84 (2.10, 3.80)	<0.001

C. All offspring

© 2023 Edlow AG et al. *JAMA Network Open*.

Variable		N		p
**Live birth during**	2018	13952	Reference	
	pandemic	18347	1.18 (0.99, 1.39)	0.060
**Maternal age (years)**		32299	1.03 (1.01, 1.04)	0.004
**Maternal race**	White	22040	Reference	
	Asian	3353	1.35 (1.04, 1.73)	0.020
	Black or African American	2955	0.73 (0.51, 1.01)	0.064
	Other	3049	1.54 (1.08, 2.17)	0.015
	Unknown	902	1.11 (0.63, 1.82)	0.698
**Maternal ethnicity**	Not Hispanic	26497	Reference	
	Hispanic	4649	0.98 (0.70, 1.35)	0.903
	Unavailable	1153	0.76 (0.44, 1.21)	0.273
**Maternal public insurance**	No	25996	Reference	
	Yes	6303	1.02 (0.80, 1.30)	0.859
**Delivery hospital type**	Academic medical center	19761	Reference	
	Community hospital	12538	0.39 (0.31, 0.48)	<0.001
**Offspring sex**	Female	15856	Reference	
	Male	16443	1.28 (1.08, 1.51)	0.004
**Pre-term birth**	No	28888	Reference	
	Yes	3411	2.63 (2.16, 3.18)	<0.001

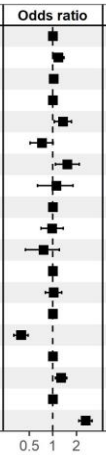

© 2023 Edlow AG et al. *JAMA Network Open*.

eFigure 6. Multiple Logistic Regression Forest Plot of Neurodevelopmental Diagnosis Within 12 Months in Offspring Associated With Period of Birth (Pandemic vs 2019)

A. Male offspring

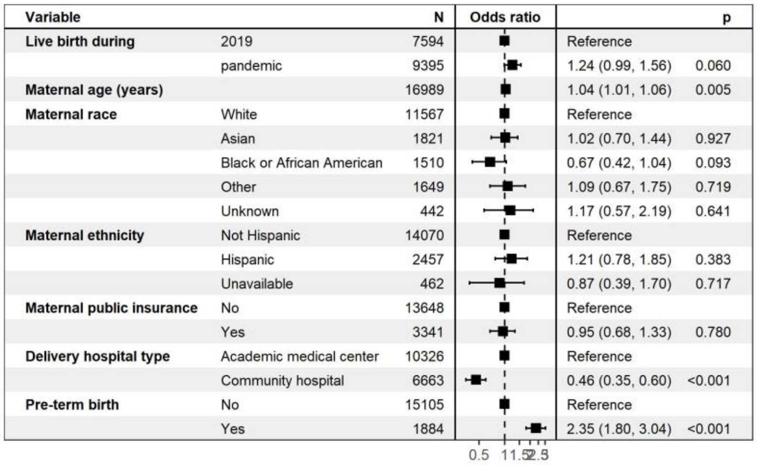

Variable		N	Odds ratio		p
Live birth during	2019	7594		Reference	
	pandemic	9395		1.24 (0.99, 1.56)	0.060
Maternal age (years)		16989		1.04 (1.01, 1.06)	0.005
Maternal race	White	11567		Reference	
	Asian	1821		1.02 (0.70, 1.44)	0.927
	Black or African American	1510		0.67 (0.42, 1.04)	0.093
	Other	1649		1.09 (0.67, 1.75)	0.719
	Unknown	442		1.17 (0.57, 2.19)	0.641
Maternal ethnicity	Not Hispanic	14070		Reference	
	Hispanic	2457		1.21 (0.78, 1.85)	0.383
	Unavailable	462		0.87 (0.39, 1.70)	0.717
Maternal public insurance	No	13648		Reference	
	Yes	3341		0.95 (0.68, 1.33)	0.780
Delivery hospital type	Academic medical center	10326		Reference	
	Community hospital	6663		0.46 (0.35, 0.60)	<0.001
Pre-term birth	No	15105		Reference	
	Yes	1884		2.35 (1.80, 3.04)	<0.001

B. Female offspring

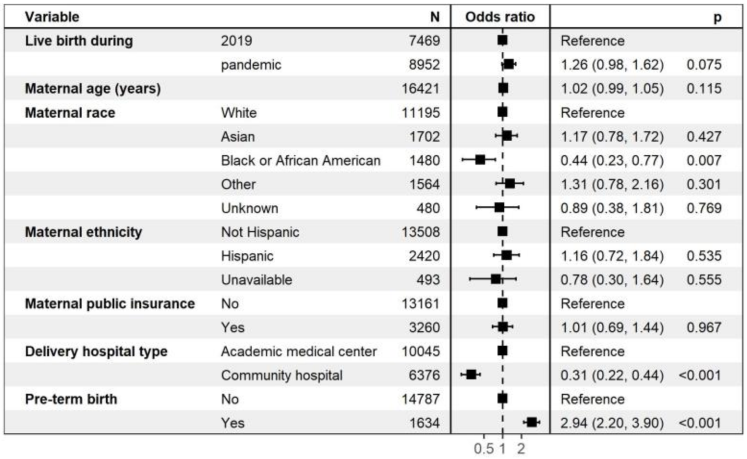

Variable		N	Odds ratio		p
Live birth during	2019	7469		Reference	
	pandemic	8952		1.26 (0.98, 1.62)	0.075
Maternal age (years)		16421		1.02 (0.99, 1.05)	0.115
Maternal race	White	11195		Reference	
	Asian	1702		1.17 (0.78, 1.72)	0.427
	Black or African American	1480		0.44 (0.23, 0.77)	0.007
	Other	1564		1.31 (0.78, 2.16)	0.301
	Unknown	480		0.89 (0.38, 1.81)	0.769
Maternal ethnicity	Not Hispanic	13508		Reference	
	Hispanic	2420		1.16 (0.72, 1.84)	0.535
	Unavailable	493		0.78 (0.30, 1.64)	0.555
Maternal public insurance	No	13161		Reference	
	Yes	3260		1.01 (0.69, 1.44)	0.967
Delivery hospital type	Academic medical center	10045		Reference	
	Community hospital	6376		0.31 (0.22, 0.44)	<0.001
Pre-term birth	No	14787		Reference	
	Yes	1634		2.94 (2.20, 3.90)	<0.001

C. All offspring

© 2023 Edlow AG et al. *JAMA Network Open*.

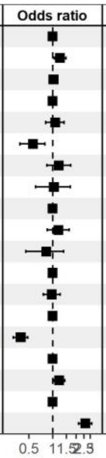

Variable		N	Odds ratio	p
**Live birth during**	2019	15063	Reference	
	pandemic	18347	1.24 (1.05, 1.47)	0.010
**Maternal age (years)**		33410	1.03 (1.01, 1.05)	0.002
**Maternal race**	White	22762	Reference	
	Asian	3523	1.08 (0.82, 1.40)	0.569
	Black or African American	2990	0.57 (0.39, 0.80)	0.002
	Other	3213	1.20 (0.84, 1.69)	0.313
	Unknown	922	1.04 (0.61, 1.68)	0.878
**Maternal ethnicity**	Not Hispanic	27578	Reference	
	Hispanic	4877	1.18 (0.85, 1.62)	0.305
	Unavailable	955	0.83 (0.46, 1.38)	0.504
**Maternal public insurance**	No	26809	Reference	
	Yes	6601	0.98 (0.76, 1.25)	0.870
**Delivery hospital type**	Academic medical center	20371	Reference	
	Community hospital	13039	0.39 (0.32, 0.49)	<0.001
**Offspring sex**	Female	16421	Reference	
	Male	16989	1.21 (1.03, 1.43)	0.024
**Pre-term birth**	No	29892	Reference	
	Yes	3518	2.59 (2.13, 3.14)	<0.001

© 2023 Edlow AG et al. *JAMA Network Open*.
