# Supplementary material for: Sex-Specific Neurodevelopmental Outcomes Among Offspring of Mothers With SARS-CoV-2 Infection During Pregnancy
Source: JAMA Netw Open. 2023 Mar 23;6(3):e234415. doi: 10.1001/jamanetworkopen.2023.4415 (PMC10037162; doi:10.1001/jamanetworkopen.2023.4415)
Supplement: Supplement 2. — Data Sharing Statement [file jamanetwopen-e234415-s002.pdf]

Data Sharing Statement

Edlow. Sex-Specific Neurodevelopmental Outcomes Among Offspring of Mothers With SARS-CoV-2 Infection During Pregnancy. *JAMA Netw Open*. Published March 23, 2023. doi:10.1001/jamanetworkopen.2023.4415

Data
Data available: No

Additional Information
Explanation for why data not available: IRB does not allow distribution of EHR data
